# Aerial attack strategies of hawks hunting bats, and the adaptive benefits of swarming

**DOI:** 10.1093/beheco/araa145

**Published:** 2021-03-31

**Authors:** Caroline H Brighton, Lillias Zusi, Kathryn A McGowan, Morgan Kinniry, Laura N Kloepper, Graham K Taylor

**Affiliations:** 1 Department of Zoology, University of Oxford, OxfordUK; 2 Department of Biological Sciences, 100 Galvin Life Science Center, Notre Dame, IN, USA

**Keywords:** attack abatement, *Buteo swainsoni*, confusion effect, dilution effect, predator–prey interaction, swarming, *Tadarida brasiliensis*

## Abstract

Aggregation can reduce an individual’s predation risk, by decreasing predator hunting efficiency or displacing predation onto others. Here, we explore how the behaviors of predator and prey influence catch success and predation risk in Swainson’s hawks *Buteo swainsoni* attacking swarming Brazilian free-tailed bats *Tadarida brasiliensis* on emergence. Lone bats including stragglers have a high relative risk of predation, representing ~5% of the catch but ~0.2% of the population. Attacks on the column were no less successful than attacks on lone bats, so hunting efficiency is not decreased by group vigilance or confusion. Instead, lone bats were attacked disproportionately often, representing ~10% of all attacks. Swarming therefore displaces the burden of predation onto bats outside the column—whether as isolated wanderers not benefitting from dilution through attack abatement, or as peripheral stragglers suffering marginal predation and possible selfish herd effects. In contrast, the hawks’ catch success depended only on the attack maneuvers that they employed, with the odds of success being more than trebled in attacks involving a high-speed stoop or rolling grab. Most attacks involved one of these two maneuvers, which therefore represent alternative rather than complementary tactics. Hence, whereas a bat’s survival depends on maintaining column formation, a hawk’s success does not depend on attacking lone bats—even though their tendency to do so is sufficient to explain the adaptive benefits of their prey’s aggregation behavior. A hawk’s success instead depends on the flight maneuvers it deploys, including the high-speed stoop that is characteristic of many raptors.

Swarming bats emerging from a massive desert roost reduce their predation risk by maintaining tight column formation, because the hawks that predate them attack peripheral stragglers and isolated wanderers disproportionately. Whereas a bat’s predation risk depends on maintaining its position within the column, the catch success of a hawk depends on how it maneuvers itself to attack, and is maximized by executing a high-speed dive or rolling grab maneuver.

## BACKGROUND

Flocking, shoaling, and swarming behaviors can all serve to reduce an individual’s predation risk, by either 1) displacing the burden of predation onto others within or outside the group; or 2) decreasing predator hunting efficiency (Krause 1994; Rieucau et al. 2015; Lehtonen and Jaatinen 2016). The first category of mechanisms encompasses the distinct phenomena of dilution, selfish herding, and marginal predation. The former is often attributed to the simple numerical dilution of per-attack risk among the individuals within a group (Foster and Treherne 1981; Morgan and Godin 1985), but group members will only enjoy a reduction in predation risk if the predator’s attack rate increases less than proportionally with group size (Turner and Pitcher 1986; Wrona and Dixon 1991). The net effect is then to displace the burden of predation onto lone individuals or smaller groups, in a phenomenon known as attack abatement. Analogous effects apply within a group, where widely spaced individuals have a larger domain of danger than tightly spaced individuals, so are expected to be attacked more often if predators attack whichever prey is closest (Hamilton 1971). Likewise, individuals at the periphery have a domain of danger extending outward from the group, so are expected to suffer higher attack rates than those in the centre (Hamilton 1971). These closely related phenomena are known as selfish herding and marginal predation, respectively, and are widely observed across taxa (Duffield and Ioannou 2017; Ioannou et al. 2017; Rayor and Uetz 1990); but see (Parrish 1989).

In contrast, the second class of mechanisms decreasing individual predation risk does so by reducing predator hunting efficiency. Within this category fall, the shared benefits of group vigilance (Lima 1995) and the confusion effect occurring when the presence of multiple prey make it harder for a predator to target any one individual (Landeau and Terborgh 1986; Quinn and Cresswell 2006; Duffield and Ioannou 2017). However, whereas confusion may well impact the outcome of a directed chase, a predator lunging or plunging into a dense prey aggregation need not be targeting any one individual. In such cases, hunting may actually be more efficient against a denser group of prey. This is true, for example, of large pelagic predators such as baleen whales that exploit the shoaling of their prey during engulfment (Cade et al. 2020), and may also be true of raptorial predators that have more opportunities to capture individual prey items when striking at a dense shoal (Nottestad and Axelsen 1999). It is an open question whether and how the confusion effect may operate in other ecological contexts.

Understanding the detailed behavior of predators is therefore essential to understanding the ecology and evolution of their interactions with prey (Lima 2002; Hein et al. 2020). For instance, the dynamics of collective motion in schooling fish is not merely influenced by their immediate response to predators (Magurran and Pitcher 1987; Handegard et al. 2012; Cade et al. 2020), but is governed by attraction and orientation rules that may themselves have evolved to promote the formation of coherent mobile groups causing cognitive or sensory confusion in predators (Ioannou et al. 2012). Likewise, hunting mode has been found to influence the group size dependence of attack frequency and catch success in raptors attacking flocking waders, leading to conflicting selection pressures on group size according to which predator species are present (Cresswell and Quinn 2010). Even so, it remains extremely difficult to make detailed, repeatable behavioral observations of the kind needed to populate detailed empirical models of predator–prey interactions—and particularly to do so in their natural ecological context, rather than in laboratory settings.

Nowhere is the challenge of making field observations clearer than in the case of predators attacking massive three-dimensional prey aggregations. The best examples to date have come from sonar studies of pelagic predators attacking schooling fish (Similä 1997; Nottestad and Axelsen 1999; Axelsen et al. 2001; Gerlotto et al. 2006; Handegard et al. 2012), and from videographic studies of aerial predators attacking murmurating birds (Carere et al. 2009; Procaccini et al. 2011; Storms et al. 2019). The former have successfully related predator attack behavior to shoal size, shape, and density, but have not yet allowed the individual outcomes of these behaviors to be observed (Handegard et al. 2012); the latter have been able to record individual outcomes, but have focused on the dynamics of the prey’s collective motion, rather than the dynamics of the predator’s attack (Carere et al. 2009; Procaccini et al. 2011; Storms et al. 2019). Hence, there are few good examples of empirical studies relating the outcomes of attacks on massive three-dimensional prey aggregations to the individual behaviors of predators and prey.

Here, we analyze the behavioral ecology of Swainson’s hawks (*Buteo swainsoni*) depredating massive swarms of Brazilian free-tailed bats (*Tadarida brasiliensis*) emerging from their roost. We test first whether maintaining column formation reduces the predation risk of individual bats, and if so whether this adaptive benefit is attributable to: 1) mechanisms that displace the burden of predation onto other individuals (i.e., attack abatement or marginal predation, possibly coupled with selfish herd effects); and/or 2) mechanisms that decrease predator hunting efficiency (i.e., vigilance or confusion effects). Second, we test whether bats are more likely to be captured in attacks from above and behind, as would be expected if vigilance were an important feature of the system, given their forward-facing eyes and sonar (Lima and O’Keefe 2013). Third, we test whether stooping dives result in higher catch success than low-speed attacks involving level flight, as predicted by a recent physics-based simulation study (Mills et al. 2018) which showed that catch success is maximized in a high-speed stoop because of the enhanced maneuverability conferred by the higher aerodynamic forces and lower roll inertia that are experienced in high-speed flight with the wings tucked. We thereby provide a full adaptive account of the predation of massive three-dimensional swarms from the distinct but interacting perspectives of predator and prey.

## METHODS

**Figure 1:**
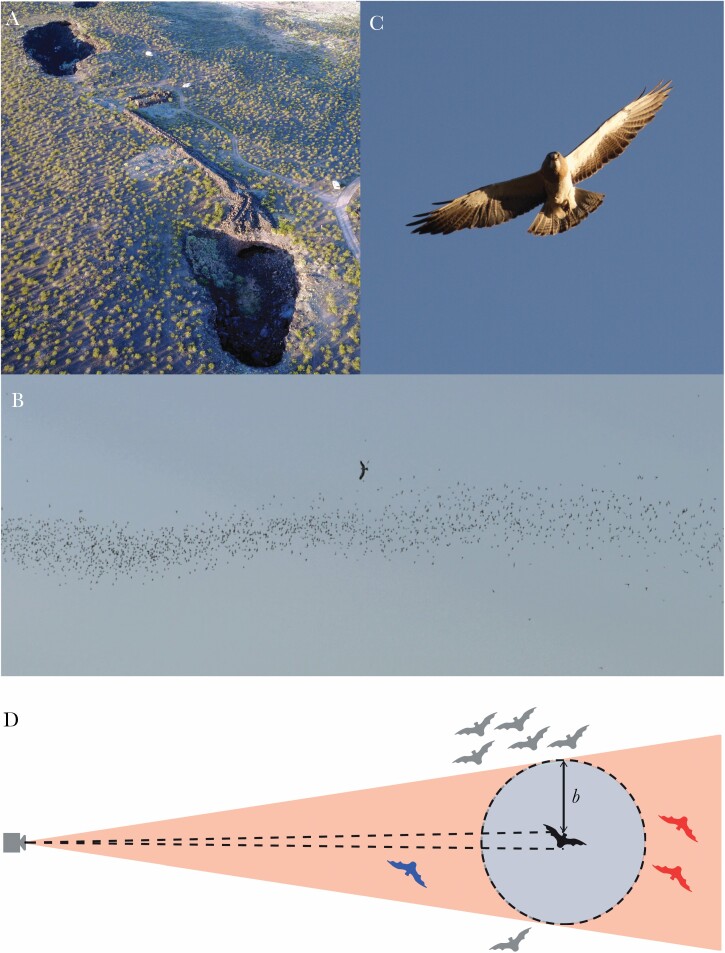
Study overview. (A) Aerial view of the Jornada caves. (B,C) Swainson’s hawk hunting Mexican free-tailed bats at the caves. Note the column formation of the swarm in (B), with only a small proportion of bats flying alone. (D) Projective geometry of a camera imaging distant targets, showing that bats (gray) falling >*b* apparent body lengths in pixels from the focal bat (i.e., outside the red frustrum) must be >*b* metric body lengths from the focal bat (i.e., outside the blue circle). The converse does not apply, because bats flying in front of (blue) or behind (red) the focal bat (black) can appear to be <*b* apparent body lengths in pixels from the focal bat (i.e., within the red frustrum) while actually being >*b* metric body lengths from the focal bat (i.e., outside the blue circle). The use of an apparent distance criterion for identifying lone bats is therefore expected to produce some false negatives, but no false positives.

We observed Swainson’s hawks hunting swarming Brazilian free-tailed bats at the Jornada Caves, New Mexico, USA. This remote site is located on a lava field, where a collapsed lava tube forms a connected pair of caves (Figure 1A). The caves are home to a maternal colony of 700,000 to 900,000 individuals (Kloepper et al. 2016), which migrate to the area during their natal season from May to September. The bats roost during the day, emerge before dusk to fly to their feeding grounds, and return individually or in small groups toward dawn. The emerging bats form a dense horizontal column (Figure 1B) which climbs away from the cave, giving the appearance first of a rising plume of smoke, then of distant clouds as the swarm splits into smaller groups on different vanishing bearings. The local population of Swainson’s hawks (Figure 1C) hunts the emerging bats daily. The bats are subject to depredation by great horned owls (*Bubo virginianus*) within the cave mouth, but the only other aerial predation events that we witnessed over three field seasons involved a single peregrine falcon (*Falco peregrinus*) hunting on three consecutive evenings in 2018.

### Behavioral observations

We systematically recorded the hawks’ hunting behavior on 15 evenings from 01 June 2018 to 24 June 2018, having witnessed the same behaviors in 2016 and 2017. We began by observing the hawks from makeshift hides, but phased these out as the birds became habituated to our presence. Emergence began between 18:19 h and 19:52 h, and hence well before sunset, which was between 20:13 h and 20:21 h. The timing of the bats’ emergence was quite variable, but the hawks usually appeared within a few minutes of its onset, suggesting that they must have been watching the caves from a distance. The number of hawks varied through the observation period, peaking at ~20. Each emergence lasted from 10 to 25 min, and we occasionally observed a second emergence from the same cave. We conducted focal follows using a voice recorder to document real-time observations made through 8 × 4 binoculars, or used a Lumix DMC-FZ1000/2500 camera to record video for later analysis (Panasonic Inc., Osaka, Japan; 1920 × 1080 pixels; 50 fps). Each observer aimed to document the entire hunting bout of a single hawk, defined as the interval from the hawk’s first appearance to its final departure. Each hunting bout comprised one or more attacks, where an attack is defined as a period of directed flight culminating in one or more grab maneuvers, defined as a motion involving rapid leg extension toward a bat.

We measured wind speed using a Kestrel 4500 Pocket Weather Tracker (Nielsen-Kellerman, PA, USA), and used the NOAA Solar Calculator (National Oceanic and Atmospheric Administration U.S. Department of Commerce 2018) to determine the time of emergence relative to sunset. We controlled for these environmental variables when testing the behavioral factors affecting capture outcome, on the basis that catch success might be expected to vary with wind speed and light level. We did not include wind direction in our analysis, because of the complications of including circular statistics in our autoregressive logistic regression models, and because we had no specific reason to expect an influence of wind direction.

### Behavioral classification

**Figure 2:**
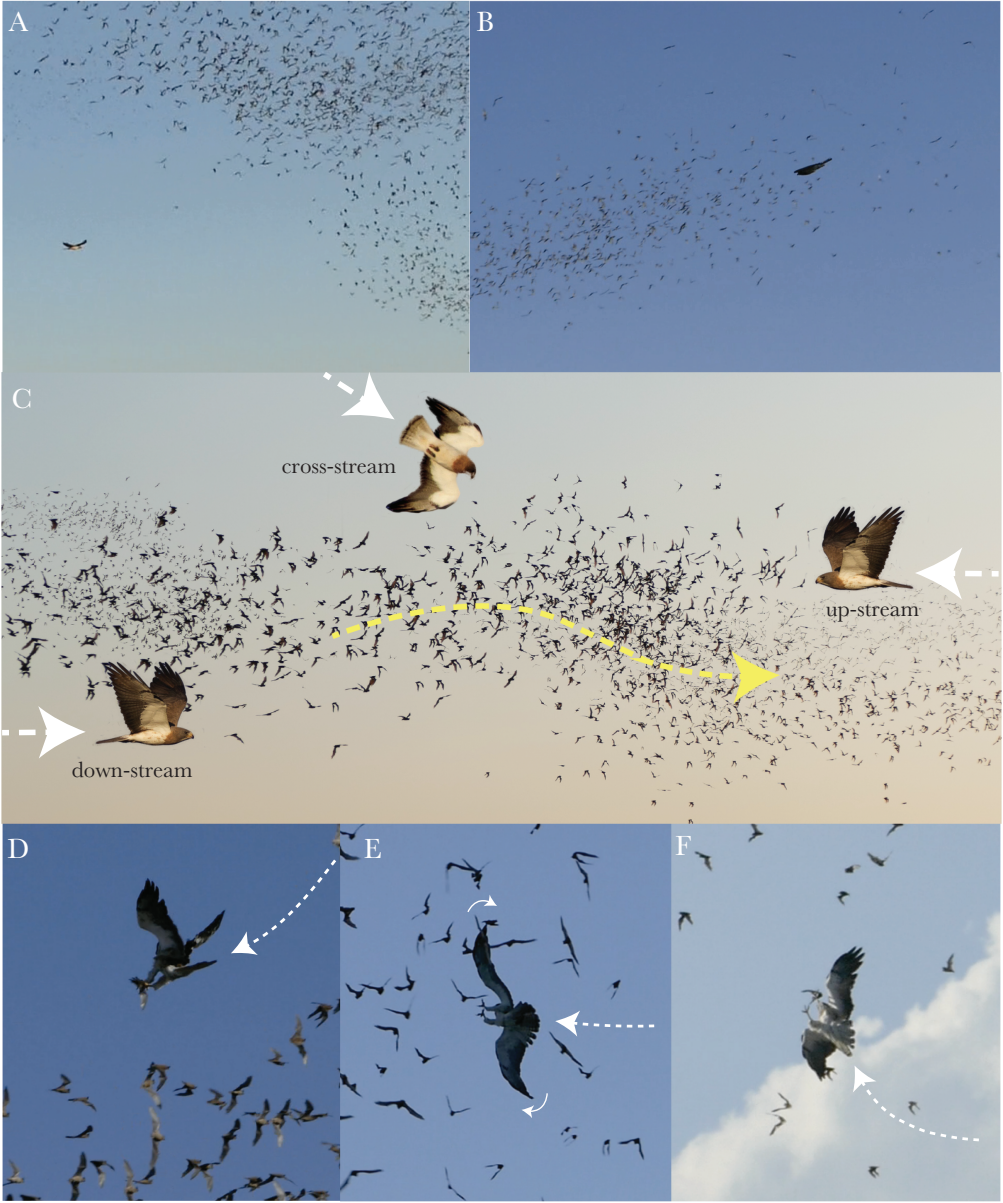
Examples of categorized attack behaviors. (A,B) Approach type, showing: (A) level flapping flight toward the column; (B) stooping dive into column, with tucked wings. (C) Approach direction, with composite image comprising video frame of swarm moving from left to right, superimposed with separate images of Swainson’s hawks to illustrate upstream, downstream, and cross-stream approach. (D–F) Grab direction, with video frames showing: (D) grab from above bat: bird extending feet downwards in a pitch-down maneuver; (E) grab from beside bat: bird extending feet horizontally in a roll maneuver; (F) grab from below bat: bird extending feet upwards in a pitch-up maneuver. White arrows indicate approximate attack trajectory. See Supplementary Movie S1 for video examples.

**Figure 3:**
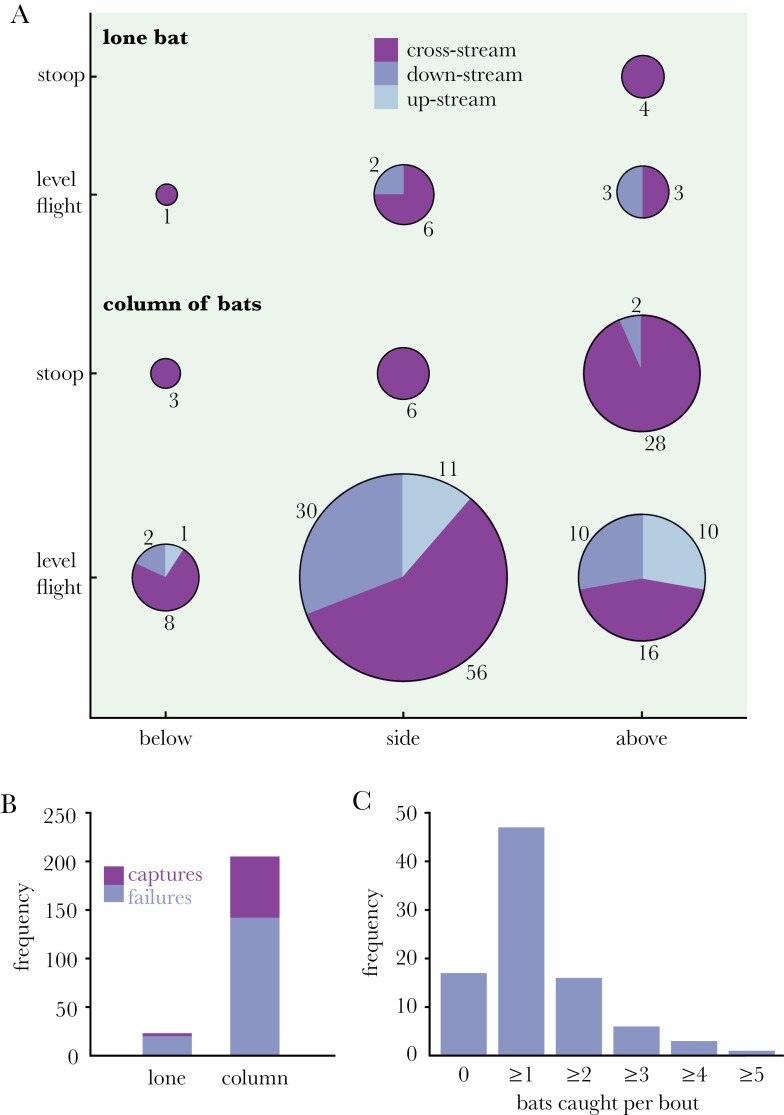
Behavioral strategies of bat-hunting Swainson’s hawks. (A) Pie charts showing frequency of each combination of behaviors for all 202 fully classified attacks; total area of each pie chart proportional to total number of observations shown by each segment. (B) Bar chart showing number of successes and failures for all *n* = 239 attacks, grouped by target type. (C) Frequency distribution of number of bats caught per hunting bout.

We categorized each attack according to: 1) approach type: level flight, or stooping dive; 2) approach direction: downstream, cross-stream, or upstream relative to bat(s); 3) grab direction: above, beside, or below targeted bat; 4) target type: lone bat, or column; and 5) capture outcome: success or failure; see Table 1, Figure 2, Supplementary Figure S1 and Movie S1. A single attack could sometimes involve more than one attempted grab if the earlier grab(s) had been unsuccessful, in which case we only recorded the direction and outcome of the final grab. We thereby estimate the success rate per attack, rather than the success rate per grab. In total, we observed the outcomes of *N* = 239 attacks from *n* = 64 hunting bouts lasting 2 h 50 m (Figure 3B), of which *N* = 202 attacks could be classified fully (Figure 3A; Supplementary Table S2; Supplementary Data S1).

**Table 1:** System used to classify hawk attack behaviors

Approach type	*Level flight*: flapping or gliding along a shallow flight path (Figure 2A)
	*Stooping dive*: fast descent on tucked wings, along a steep dive path (Figure 2B)
Approach direction	*Downstream*: hawk flying in same direction as bat(s) (Figure 2C)
	*Upstream*: hawk flying in opposite direction to bat(s) (Figure 2C)
	*Cross-stream*: hawk flying in any other direction relative to bat(s) (Figure 2C)
Grab direction	*Above*: grab initiated from above bat, in a pitch-down maneuver with the legs extended downwards (Figure 2D)
	*Beside*: grab initiated from beside bat, in a roll maneuver with the legs extended horizontally (Figure 2E)
	*Below*: grab initiated from beneath bat, in a pitch-up maneuver with the legs extended upwards (Figure 2F)
Targeting strategy	*Lone bat*: attack on bat flying >5 body lengths from the edge of the column, or in a different direction to the column (Supplementary Figure S2C–F)
	*Column of bats*: attack on one or more bats flying within a cohesive group of individuals, all flying in the same general direction (Supplementary Figure S2A,B)
Capture outcome	*Success*: bat caught, independent of whether subsequently dropped or eaten
	*Failure*: bat not caught

We aimed to classify a targeted individual as a lone bat if it was either an isolated wanderer or a straggler flying on the periphery of the column, thereby including bats that were unable to benefit from dilution through attack abatement, or that were subject to marginal predation and possible selfish herd effects. We follow the recommended practice of using an absolute distance criterion and a behavioral difference criterion to assess group membership (Stankowich 2003). Targeted individuals were recorded as lone bats if they were judged to be flying outside the main body of the column, or appeared to be flying in a different direction to its coordinated members. Consistent with a convention established in the literature on fish schooling (Stankowich 2003), those individuals that we classified as lone bats on a distance criterion were always >5 body lengths from their nearest neighbor, but were typically more distant than this. Under the projective geometry of an imaging system located far from its subjects (Figure 1D), any individual appearing to be >*b* body lengths in pixels from its nearest neighbor in the image must also be >*b* metric body lengths from its nearest neighbor in space. The converse does not apply, however, because an individual that is >*b* metric body lengths from its nearest neighbor may not appear so if silhouetted against the column (Figure 1D), so our identification of lone bats is expected to produce some false negatives but no false positives (see Section 2D). Classifying individuals as lone bats if they were flying in a different direction to the swarm therefore proved useful in identifying stragglers that were silhouetted against the column, and wanderers that were flying in uncoordinated proximity to other individuals.

### Proportion of lone bats

We estimated the probability that a bat drawn at random from the population was flying alone, *P*(L), or in the column, *P*(C), by estimating their sample proportions $\left(\hat{p}\right)$ (L) and $\left(\hat{p}\right)$ (C) in a representative sample of 18 video frames (Supplementary Figure S2). For each frame, we counted the number of bats meeting the criteria for classification as lone bats (Section 2B). We then estimated the total number of bats automatically by using the count function in Adobe Photoshop CC2019, having binarized each image using a threshold just sufficient to make the background entirely white. Sample frames were chosen as meeting the following requirements: 1) each frame recorded during a separate attack, to avoid pseudo-replication; 2) camera zoomed out and in focus, to maximize coverage; 3) column formation clearly visible, to visualize margins; 4) bats close enough to the camera to see their wings, to allow identification; and 5) background composed entirely of sky, to enable automation. Because our observations were made as the bats had streamed out of the cave mouth, lone bats remained within the same sector of sky as the column, so their count should not have been significantly biased by them drifting out of frame.

### Theoretical framework

The relative risk of predation for a lone bat versus a bat flying in the column is defined as $RR_{L}^{S}=P(S|L)/P(S|C),$ where *P*(S|L) and *P*(S|C) measure the risk of successful capture to a bat flying alone or in the column. Neither quantity can be estimated directly, but we may use Bayes’ theorem to rewrite the relative risk of predation as:


$$\begin{array}{l} RR_{L}^{S}=\frac{P(L|S)}{P(C|S)}\times \frac{P(C)}{P(L)} \end{array}$$ (1)

which multiplies the odds that a successfully captured bat is flying alone, *O*_L|S_ = *P*(L|S)/*P*(C|S), by the odds that a bat drawn at random from the population is flying in the column *O*_C_ = *P*(C)/*P*(L). These odds are readily estimated from our behavioral observations (Section 2B) and from our representative sample of video frames (Section 2C). Equation 1 measures the net disadvantage experienced by lone bats relative to bats flying in the column, and may be written compactly as $RR_{L}^{S}=O_{L|S}\times O_{C},$ which can be used to assess whether flying in the column reduces an individual’s predation risk (i.e., if $RR_{L}^{S}>1$).

To separate the causes of why $RR_{L}^{S}>1$ into the two categories of mechanism defined in the Introduction, we return to the original definition of the relative risk of predation, $RR_{L}^{S}=P(S|L)/P(S|C).$ Applying the chain rule, and using *P*(A) to denote the probability of an attack, we have *P*(S∩L) = *P*(S|L)*P*(L) and *P*(A∩S∩L) = *P*(S|LA)*P*(L|A)*P*(A), where *P*(L|A) is the probability that an attacked bat is flying alone, and *P*(S|LA) is the probability that an attack on a lone bat is successful. Because the occurrence of a capture implies that an attack has occurred, it follows that *P*(S∩L) = P(A∩S∩L), and hence that *P*(S|L) = *P*(S|LA)*P*(L|A)*P*(A)/*P*(L). Similarly, we may write *P*(S|C) = *P*(S|CA)*P*(C|A)*P*(A)/*P*(C). Dividing *P*(S|L) by *P*(S|C), we can therefore express the relative risk of predation for a lone bat as:


$$\begin{array}{l} RR_{L}^{S}=\frac{P(L|A)P(C)}{P(C|A)P(L)}\times \frac{P(S|LA)}{P(S|CA)} \end{array}$$ (2)

The first term on the righthand side of Equation 2 is just the relative risk of attack for a lone bat versus a bat flying in the column (*cf.* Equation 1), which we will denote $RR_{L}^{A}$. The second term is just the relative risk of successful capture in an attack on a lone bat versus an attack on the column, which we will denote $RR_{LA}^{S}$. Equation 2 may therefore be written compactly as $RR_{L}^{S}=RR_{L}^{A}\times RR_{LA}^{S},$ where $RR_{L}^{A}$ and $RR_{LA}^{S}$ are both readily estimated quantities.

The relative risk of attack, $RR_{L}^{A}=O_{L|A}\times O_{C},$ measures the net disadvantage that lone bats experience as a result of any mechanism that displaces the burden of predation from the column and onto them—whether because they are isolated wanderers (i.e., individuals unable to benefit from dilution through attack abatement) or stragglers on the periphery (i.e., individuals subject to marginal predation, and perhaps selfish herd effects). In contrast, the relative risk of capture in an attack, $RR_{LA}^{S}=P\left(S\;|\;LA\right)/P(S|CA),$ measures the net disadvantage that lone bats experience as a result of not benefitting from any mechanism decreasing predator hunting efficiency in attacks on the column (i.e., group vigilance or confusion effects). Hence, if lone bats do indeed have a high relative risk of predation (i.e., if $RR_{L}^{S}>1$), then Equation 2 can be used to assess whether this is attributable to a higher risk of attack (i.e., if $RR_{L}^{A}>1$), or a higher risk of capture if attacked (i.e., if $RR_{LA}^{S}>1$).

Identifying individuals in a dense moving aggregation is always challenging, but is particularly so in three dimensions. For example, some of the attacks categorized as attacks on the column will actually have been attacks on lone bats silhouetted against the column (Section 2B), leading us to underestimate the odds *O*_L|S_ and *O*_L|A_. Likewise, individual bats with overlapping silhouettes would have been counted as one within the column (Section 2C), leading us to underestimate the odds *O*_C_. It follows that our estimates of the relative risk of predation, ${\hat{RR}}_{L}^{S}={\hat{o}}_{L|S}\times {\hat{o}}_{C},$ and the relative risk of being attacked, ${\hat{RR}}_{L}^{A}={\hat{o}}_{L|A}\times {\hat{o}}_{C},$ will both tend to understate the adaptive benefits of maintaining column formation, making us conservative in the conclusions that we draw.

### Statistical analysis

We use the theoretical framework developed in Section 2D to provide an empirical account of the bats’ swarming behavior, through a statistical analysis of sample odds $\left(\hat{o}\right)$ and sample proportions $\left(\hat{p}\right)$. This is complemented by an analysis of the factors affecting catch success, which we use to develop an empirical account of the hawks’ hunting behavior. We conducted all of the statistical analysis in *R* version 3.6.1 (RCoreTeam 2019), using the packages *PropCIs* (Scherer 2018), *plyr* (Wickham 2011), and *boot* (Canty and Ripley 2019). As far as possible, the analysis is designed to account for the nonindependence of successive attacks within a hunting bout. However, as there was no way of identifying individual hawks, we were unable to control for the possibility that we sampled the same individuals repeatedly across days. Raw data and statistical code are provided in Supplementary Data S1 and Code S1.

To avoid pseudo-replication within hunting bouts, we computed a 95% profile-likelihood confidence interval (CI) for the mean attack rate *λ* over all *n* = 64 bouts using an intercept-only quasi-Poisson regression with log bout duration as an offset variable. We computed a 95% profile-likelihood CI for the mean number of bats caught per bout using an intercept-only quasi-Poisson regression with no offset variable. Adding the log number of attacks by bout as an offset variable allowed us to compute a 95% profile-likelihood CI for the overall success rate per attack, $\hat{p}(S|A)$. Results of the quasi-Poisson regression models are reported together with the corresponding dispersion parameter ϕ.

Hawks with a lower individual success rate may have made a greater number of attacks, thereby contributing disproportionately to the sample used to calculate $\hat{p}(S|A),$ so we also report the mean success rate per attack averaged by hunting bout, defined as $\bar{p}\left(S|A\right)=\frac{1}{n}\underset{i=1}{\overset{n}{\sum }}\;{\hat{p}}_{i}(S|A)$ where ${\hat{p}}_{i}(S|A)$ denotes the success rate per attack for the *i*th hunting bout. We report $\bar{p}\left(S|A\right)$ together with a bias-corrected and accelerated bootstrap 95% CI (Efron 1987), computed using stratified resampling over 10^6^ resamples (Davison and Hinkley 1997). Other sample proportions or odds were computed directly from the data, and are reported together with a 95% Wilson score CI. These score CIs are expected to have less than nominal coverage probability because of the nonindependence of attacks from the same hunting bout, but we report them in preference to stating only a point estimate of the sample odds or proportions.

The first-order autocorrelation coefficient (*r*_1_ = 0.239) for the outcomes of the *N* = 175 attacks preceded by another from the same hunting bout fell well outside the 95% CI for white noise (−0.148, 0.148), so we used autoregressive (AR) logistic regression to test the factors affecting attack outcome. To avoid having to discard the first attack of each bout in our autoregressions, we concatenated the time series data for consecutive hunting bouts, which effectively substitutes white noise for the autoregressive term corresponding to the first attack of each bout. Provisional model-order selection using the Akaike Information Criterion (AIC) in a pure autoregressive model of capture outcome supported use of a first-order AR(1) model. We similarly used AR(1) logistic regression to test for a relationship between target type and attack behavior. We used likelihood ratio tests to assess the significance of the model factors at *α* = 0.05, Wald tests to compute the significance of differences between levels, and profile-likelihood 95% CIs to quantify the uncertainty in the parameter estimates. Odds ratios from the logistic regressions were computed by exponentiating the logistic regression coefficients.

## RESULTS

### Bat-hunting hawks achieve high catch success at high hunting intensity

The *n* = 64 hunting bouts that we observed lasted from 20 to 726 s (median: 120 s; first, third quartiles: 68, 206 s), and most involved multiple attacks (median: 3; first, third quartiles: 2, 5; maximum: 15) made at high hunting intensity (*λ* = 0.0234 s^−1^; CI: 0.0175, 0.0304 s^−1^; ϕ=4.66; df = 63). Three-quarters of the observed hunting bouts resulted in the capture of at least one bat (75.0%; CI: 63.2%, 84.0%), and as the hawks invariably consumed their prey on the wing, they were often able to catch more than one bat per hunting bout (mean: 1.16; CI: 0.92, 1.43; ϕ=0.97; df = 63; Figure 3C). Bats were caught at an overall success rate per attack of $\hat{p}(S|A)=0.310$ (CI: 0.241, 0.390; ϕ=1.11; df = 63), but the mean success rate per attack was significantly higher when averaged by hunting bout, at $\bar{p}\left(S|A\right)=0.434$ (bootstrap CI: 0.395, 0.475; *n* = 64 bouts). This implies that individuals with a higher success rate made fewer attacks—presumably because they reached satiation sooner. Hence, whereas $\hat{p}(S|A)$ provides the best estimate of the probability that an attack ends in a successful capture, $\bar{p}\left(S|A\right)$ provides a better estimate of the hunting efficiency of a hawk drawn at random from the population.

### Flying in the column reduces predation risk in swarming bats

Only 5% of the bats that we observed being captured were classified as lone bats ($\hat{p}(L|S)=0.045$; CI: 0.016, 0.125; *n* = 66 captures classified by target type; Figure 3B). This represents a small proportion of the total catch, but is many times higher than the proportion of the total population meeting the criteria for classification as lone bats, given that $\hat{p}(L)\approx 0.2\%$ for the >34,000 bats visible in a representative sample of 18 video frames (Supplementary Figure S2, Supplementary Table S3). To assess the significance of this result, we note that the relative risk of predation for a lone bat is $RR_{L}^{S}=O_{L|S}\times O_{C}$ (see Equation 1), where $\;{\hat{o}}_{L|S}=0.048$ is the odds that a caught bat was a lone bat (CI: 0.016, 0.143; *n* = 66 captures classified by target type). Lone bats are therefore expected to experience a higher overall predation risk than bats flying in the column (i.e., $RR_{L}^{S}>1$) if *O*_*C*_ ≥ 21 (CI: 7, 63). In fact, we find that $\;{\hat{o}}_{C}>500,$ which is an order of magnitude higher than the critical value of *O*_*C*_. We robustly conclude that flying in the column significantly reduces predation risk, such that lone bats have a high relative risk of predation with ${\hat{RR}}_{L}^{S}\gg 1$.

### Mechanisms reducing predation risk in the column

To identify the mechanisms underlying the reduced predation risk in the column, we rewrite the relative risk of predation as $RR_{L}^{S}=RR_{L}^{A}\times RR_{LA}^{S}$ (see Equation 2), where $RR_{L}^{A}$ denotes the relative risk of attack for a lone bat, and $RR_{LA}^{S}$ denotes the relative risk of capture for a lone bat that is attacked. Clearly, $RR_{LA}^{S}$ would have to be significantly greater than one for us to conclude that either vigilance or confusion explain the lower predation risk to which swarming bats are exposed. In fact our best estimate of $RR_{LA}^{S}$ was ${\hat{RR}}_{LA}^{S}<1$, and there was no significant difference in the hawks’ catch success for attacks on lone bats versus attacks on the column (likelihood ratio test in AR(1) logistic regression: *χ*^2^(1) = 2.582, *p* = 0.108; *n* = 228 attacks categorized by target type). It follows that there is no evidence of any vigilance or confusion effect (Figure 3B). In contrast, the relative risk of attack for a lone bat, $RR_{L}^{A}=O_{L|A}\times O_{C},$ represents the combined effects of attack abatement, marginal predation, and selfish herd effects (see Section 2D). Given that $\;{\hat{o}}_{L|A}=0.112$ (CI: 0.073, 0.172; *n* = 228 attacks categorized by target type), we require that *O*_*C*_ ≥ 9 (CI: 6, 14) to conclude that one or more of these mechanisms is occurring. Hence, since $\;{\hat{o}}_{C}>500$, we robustly conclude that ${\hat{RR}}_{L}^{A}\gg 1$ and hence that dilution through attack abatement and/or the avoidance of marginal predation (possibly coupled with selfish herd effects) is necessary and sufficient to explain the lower predation risk for bats flying in the column. To summarize, although the great majority of the hawks’ attacks were made against the column ($\hat{p}\left(C\;|\;A\right)=0.899$; CI: 0.853, 0.932), lone bats suffered a high relative risk of predation because they were disproportionately more likely to be attacked than bats flying within the column.

### Hawks attacking the column have multiple opportunities to grab a bat

Although we never observed more than one bat being captured in a single attack, presumably because of the processing required, attacks on the column could sometimes involve up to three attempted grabs if the preceding grab(s) were unsuccessful. Other things being equal, and in the absence of any significant vigilance or confusion effect, we might therefore have expected to see a higher success rate per attack against the column—consistent with the qualitative observation that ${\hat{RR}}_{LA}^{S}<1,$ albeit not significantly so. In principle, the expected catch success of an attack involving up to *k* independent grabs is *P*(S|A_*k*_) = 1 − (1 − *q*)^*k*^, where *q* represents the probability that a given grab proves successful. Treating the observed catch success against lone bats ($\hat{p}\left(S|LA\right)=0.130$; CI: 0.045, 0.321; *n* = 23 attacks on lone bats) as an estimate of *q*, the expected catch success of an attack involving up to *k* = 3 independent grabs, *P*(S|A_*k*=3_) = 0.342, is statistically indistinguishable from the overall catch success for attacks on the column ($\hat{p}\left(S|CA\right)=0.307$; CI: 0.248, 0.374; *n* = 205 attacks on the column). Hence, while we are not in a position to conclude statistically that the hawks were any more or less successful in their attacks against the column than against lone bats (Section 3C), our results are consistent with the possibility that the hawks improved their effective success rate when attacking the column by taking multiple opportunities to grab a bat.

### Stoops and rolling grab maneuvers are associated with higher catch success

**Figure 4:**
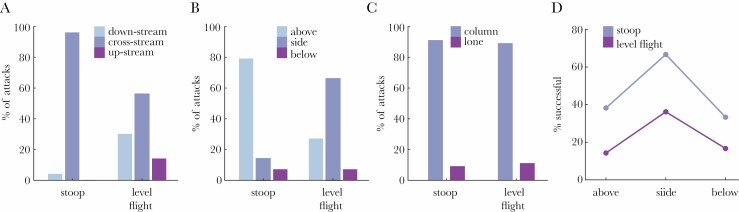
Behavioral interactions in bat-hunting Swainson’s hawks. (A–C) Proportions of attacks involving different categories of behavior in stooping versus level flight for (A) approach direction; (B) grab direction; (C) targeting strategy. (D) Interaction plot showing that using either a stooping dive or a rolling grab maneuver from the side increases catch success independently.

We used AR(1) logistic regression to model how the odds of capture were related to approach type, approach direction, grab direction, target type, wind speed, or time before sunset—of which only approach type and grab direction were significant. A reduced AR(1) model retaining only these significant factors was better supported than the full AR(1) model (∆AIC = 7.2), and was also better supported than a pure AR(1) model without any factors (∆AIC = 6.0). There was no evidence of any statistically significant interaction between approach type and grab direction (Figure 4D), because the reduced AR(1) model containing only their main effects was better supported than an extended AR(1) model also containing their interaction (∆AIC = 3.8). As predicted, the expected odds of capture in the reduced AR(1) model were 3.46 times higher (CI: 1.43, 8.82) in a stoop than in level flight (Wald test: *z* = 2.70, *P* = 0.007). Moreover, they were 3.45 times higher (CI: 1.54, 8.48) if the bat was grabbed in a rolling maneuver from the side rather than in a pitching maneuver from above (Wald test: *z* = 2.87, *P* = 0.004).

### Hawks favor alternative attack strategies maximizing catch success

The vast majority of the *n* = 218 attacks that we could categorize by approach direction involved either a cross-stream approach (65.1% of attacks; CI: 58.6%, 71.2%; Figure 3A) or a downstream approach (24.3% of attacks; CI: 19.1%, 30.4%; Figure 3A). Upstream approaches were infrequent (10.6% of attacks; CI: 7.1%, 15.3%; Figure 3A), consistent with the prediction that hawks should avoid attacking bats frontally to avoid their visual and acoustic gaze. It is also plausible that the hawks avoided frontal attacks because these are difficult or risky to execute, although we found no statistical evidence that approach direction influenced the odds of capture. Approximately one-fifth of the *n* = 202 attacks that we could categorize fully involved stooping (21.3% of attacks; CI: 16.2%, 27.4%; Figure 3A). Almost all of these *n* = 43 stoops involved a cross-stream approach (95.3% of stoops; CI: 84.5%, 98.7%; Figure 4A), and most involved a pitching grab maneuver from above (79.1% of stoops; CI: 64.8%, 88.6%; Figure 4B). In contrast, the majority of the *n* = 159 attacks made in level flight involved a rolling grab maneuver from the side (66.0% of attacks; CI: 58.4%, 72.9%; Figure 4B). Hence, the great majority of the *n* = 202 attacks that we could categorize fully involved either a stoop or a rolling grab maneuver (73.3% of attacks; CI: 66.8%, 78.9%)—these also being the only behavioral tactics that were associated with significantly improved capture odds (Section 3E). These two tactics were only rarely used in combination, however (3.0% of attacks; CI: 1.4%, 6.3%), so they appear to represent alternative rather than complementary tactics. We found no evidence that the hawks modulated their attack strategy in relation to whether they were attacking a lone bat or the column, because target type was not significantly related to approach type (Figure 4C), approach direction, or grab direction in an AR(1) logistic regression model.

## DISCUSSION

### An adaptive account of the bats’ swarming behavior

The bats’ emergence in broad daylight is presumably driven by a need to time their arrival at distant feeding grounds to coincide with the activity of their insect prey (Fenton et al. 1994), or a need to find water early given the high daytime temperatures experienced in their caves (Herreid 1963). Both challenges must be acute at the Jornada Caves, which are located on a parched lava field 16km from the closest point on the Rio Grande, separated by desert. As a result, the bats are exposed to intense predation by diurnal raptors on emergence, so it is not surprising that they return to the caves under cover of darkness, having met their daily needs for food and water. The 74 bats whose capture we have documented here over 15 days constitute only ~0.01% of the colony, but they also represent only a small fraction of the total catch. Considering that the local population of hawks sustains its hunting activity over 4–5 months, it is probable that something on the order of ~0.1% of the bat population falls victim to the hawks each year. Against such persistent predation risk, it makes sense to ask whether the collective behavior that the bats exhibit on emergence reduces their individual risk.

It was rare to see individual bats flying far from the column, so as only ~0.2% of the total population were classified as lone bats in sample images of their emergence, compared to ~5% of those that we recorded being caught, it is clear that the predation risk of lone bats must have been an order of magnitude or more higher than the predation risk of bats flying in the column $\left({\hat{RR}}_{L}^{S}\gg 1\right)$. It follows that there should be strong selection in favor of mechanisms promoting aggregation on emergence, and for as long as the hawks interact with the swarm. This may explain why this species produces echolocation calls with different acoustic characteristics to foraging calls as they depart their caves in dense column formation, which has been hypothesized to facilitate identification of spatial position within the group, or to serve in social communication (Gillam et al. 2010). Away from the central place of the cave, the expected encounter rate of lone predator and lone prey may be so low as to make the benefits of maintaining column formation negligible—or perhaps even negative, given the high visibility and long detection range of the column. This may explain why the swarm loses its coherence away from the cave, although column breakdown also appeared to be associated with collective decision making, because the resulting subgroups usually headed off on different vanishing bearings—presumably to different feeding grounds. The hawks, on the other hand, tended to remain in the vicinity of the cave, apparently reaching satiation before the bats had finished their emergence.

**Figure 5:**
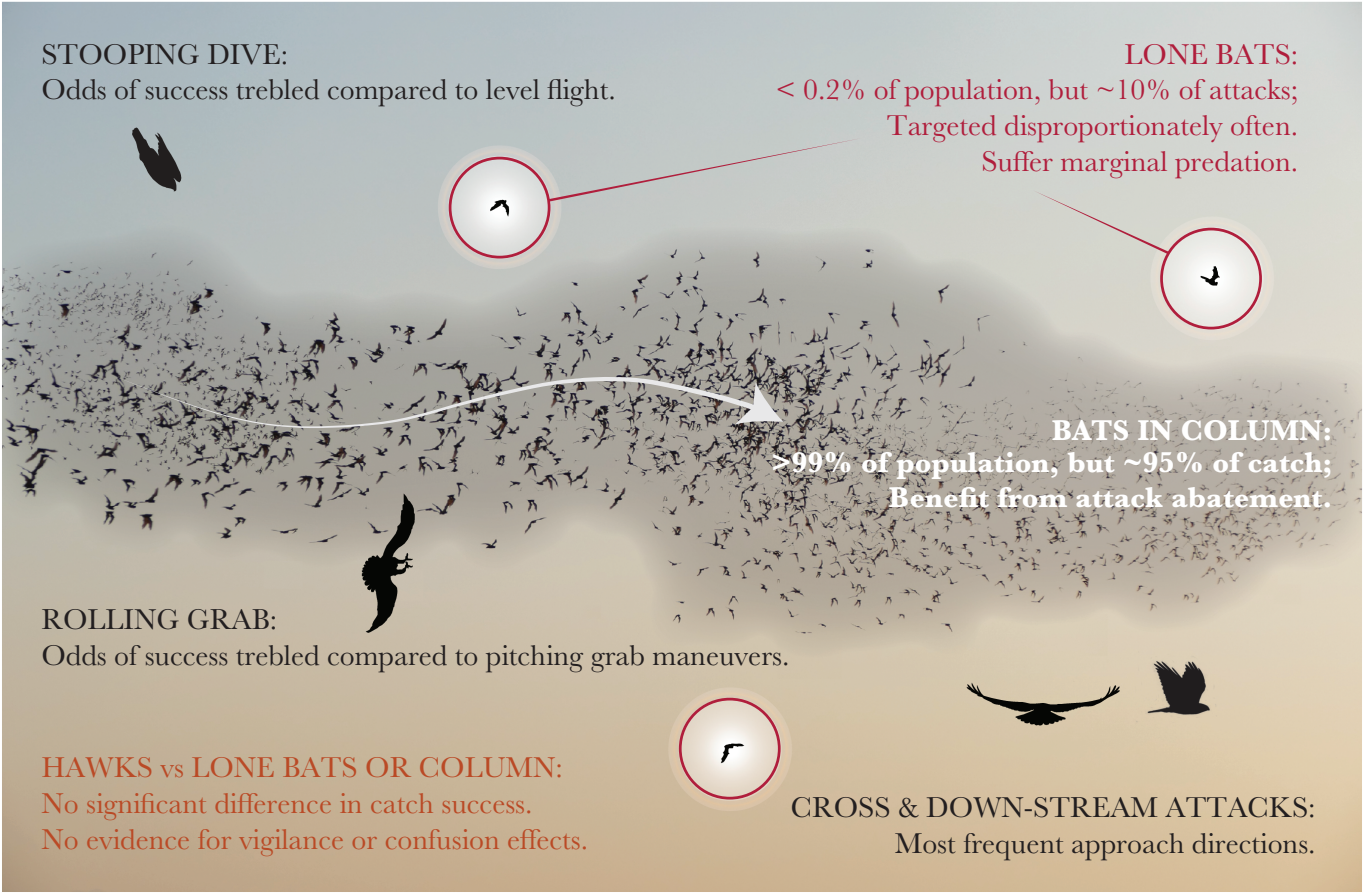
Diagrammatic representation of our key findings, showing the hunting behaviors that were associated with the highest catch success for the hawks, and the effects of flying in the column for the bats.

The observed capture rate per attack was not significantly different for bats flying alone or in the column, so we found no evidence that swarming bats benefitted from any group vigilance or confusion effect decreasing predator hunting efficiency (Figure 5). This does not exclude the possibility that bats flying within the column might still have benefitted from being able to maintain lower individual vigilance levels than lone bats, with no associated increase in predation risk. However, as the bats were commuting rather than foraging, there is no obvious opportunity cost to maintaining a high level of vigilance. Moreover, because each attack on the column could involve up to three attempted grabs if the preceding grab(s) had been unsuccessful, the hawks had multiple opportunities to catch a bat when attacking the column. This could in principle have masked any effects of vigilance or confusion depressing the capture rate per grab, but a binomial model of catch success for attacks involving multiple grabs provided no evidence that this was the case (Section 3D). On the contrary, the observed capture rate per attack was consistent with the null hypothesis that the hawks achieve the same success rate per grab in attacks involving multiple grabs at the column as in attacks involving a single grab at a lone bat.

It follows that the lower predation risk of bats flying within the column must derive not from group vigilance or confusion effects decreasing predator hunting efficiency, but from mechanisms displacing the burden of predation onto lone bats (see Introduction). This conclusion is straightforwardly confirmed by comparing the proportion of observed attacks that were attacks on lone bats (~10%) to the overall proportion of lone bats (~0.2%), which shows that the risk of being attacked must have been more than an order of magnitude higher for a lone bat than for a bat flying in the column $\left({\hat{RR}}_{L}^{A}\gg 1\right)$. Dilution through attack abatement and/or the avoidance of marginal predation (possibly coupled with selfish herd effects) is therefore both necessary and sufficient to explain the lower predation risk of bats flying within the column (Figure 5). Given these clear adaptive benefits of maintaining column formation, it remains an open question why any bat ever flew separately from it. Although it is plausible that some of these individuals might have foraged in the vicinity of the cave, instead of flying to distant feeding grounds, it seems more likely that such behavior is simply maladaptive. For example, individuals may have become detached from the column as a result of stochastic processes, perhaps biased by individual variation in sensorimotor physiology and the algorithms underpinning collective behavior (Ioannou et al. 2012).

### An adaptive account of the hawks’ hunting behavior

The tendency of the hawks to attack lone bats disproportionately often—though still less frequently than they attacked bats in the column—is consistent with the theory of attack abatement in the case of lone bats representing isolated wanderers (Turner and Pitcher 1986), and with the theory of marginal predation in the case of lone bats representing stragglers on the periphery of the column (Hamilton 1971). More generally, it is consistent with the expectation that individuals within a dense group will have a smaller domain of danger than isolated or peripheral individuals if predators target whichever prey is closest to them (Hamilton 1971; Quinn and Cresswell 2006; Duffield and Ioannou 2017). Indeed, Hamilton introduced his selfish herd theory by commenting that “with the dense and sudden emerging columns of bats that have been described issuing at dusk from great bat caves … observations that predators do often take isolated and marginal individuals have frequently been recorded.” (Hamilton 1971), although the references that he cited to support this assertion are anecdotal at best. Hamilton’s example relating to emerging bats is demonstrated formally here for the first time.

The enlarged domain of danger of isolated and peripheral individuals may already be sufficient to explain why lone bats were targeted disproportionately often, but it is also plausible that the hawks specifically targeted lone individuals when it was possible to do so. For example, targeting lone bats may present a lower risk of injury than flying through a dense swarm—whether from an ill-timed collision, or from a defensive bite—although we find no clear evidence of this, given how often the hawks plunged deep into the swarm. Formal modeling of the chase dynamics (Brighton et al. 2017; Brighton and Taylor 2019) will be required to test whether the hawks target specific individuals on approach, or whether they simply grab at whichever bat happens to be closest, having broadly targeted the swarm. In any case, we find no evidence that the hawks adopt systematically different attack strategies against lone bats versus bats flying in the column (Section 3F).

Our observations provide perhaps the first empirical evidence that stooping enhances catch success against agile prey, more than trebling the odds of success compared to an attack initiated in level flight (Section 3E; Figures 4D and 5). This result is consistent with a recent physics-based simulation study, which found that the catch success of model falcons attacking agile model prey was maximized by initiating an attack from a high-speed dive (Mills et al. 2018). In these simulations, stooping enhanced catch success through: 1) the higher aerodynamic forces available for maneuvering at higher airspeed; 2) the lower roll inertia present with the wings tucked; and 3) the speed advantage conferred in a tail chase (Mills et al. 2018). However, the Swainson’s hawks that we studied almost always used a pitching grab maneuver to catch bats when stooping, and tended to use a cross-stream approach, rather than a tail chase (Figure 5). The mechanistic benefits of stooping in this case are therefore likely to be either: 1) the higher aerodynamic forces available for maneuvering at higher airspeed; or 2) the element of surprise that stooping may confer. We saw no evidence that stooping served to split the column, and our observations provide no evidence that this would have benefited the attacker, given that attacks on lone bats were no more likely to be successful than attacks on the column.

Despite its tactical advantages, stooping was only used in 21% of the attacks that we observed (Figure 3A). This might have been because having already lost altitude in a stoop, it was more efficient to make subsequent attacks in level flight, but this is contradicted by the fact that nine of the observed hunting bouts involved repeated stoops. More importantly, attacks made from level flight could be just as successful as attacks made from stoops, provided they involved a rolling grab maneuver (Figure 4D). Other things being equal, this more than trebled the odds of catch success relative to the use of a pitching grab maneuver (Figure 5), consistent with the mechanical advantages expected when banking into a turn (Mills et al. 2018). Overall, the majority of the attacks that we observed involved a rolling grab maneuver (55% of attacks) and/or a stooping dive (21% of attacks), confirming that the hawks had a strong tendency to adopt their two most successful behavioral tactics (Section 3F). Moreover, as these tactics were rarely combined in a single attack (3% of attacks), but were deployed in quick succession on 17 of the observed hunting bouts, it would appear that they represent alternative and complementary attack strategies, rather than idiosyncratic behavioral traits of particular individuals.

### Behavioral interaction effects

Although the swarm was occasionally scattered by an attack, the only definite evasive behavior that we witnessed was a last-ditch attempt to avoid capture by an individual at immediate risk of being grabbed. In any case, given that 13% of attacks on lone bats ended in their capture, evading an attack when flying alone is evidently a less effective strategy than avoiding an attack altogether by flying in the column. This may partly reflect the challenges of predator detection: bats sense their environment using echolocation and vision, which owing to their forward-facing eyes and sonar can result in blind zones above and behind the bat (Lima and O’Keefe 2013). The relative infrequency of upstream approaches that we observed (Figures 3A and 4A) may therefore be adaptive for the hawks (Section 3F), since a downstream or cross-stream approach avoids placing the attacker within the primary visual and acoustic gaze of its target, while simultaneously reducing the demands on the attacker’s guidance and control (Mills et al. 2018). We found no evidence that the hawks modulated their hunting tactics according to whether they were attacking a bat flying alone or in the column.

### Avian predation of bats as a global selection pressure

Although there are numerous anecdotal reports of birds hunting bats (Supplementary Table S1), there have been few systematic studies of this behavior (Rodríguez-Durán and Lewis 1985; Black et al. 1979; Fenton et al. 1994; Roberts et al. 1997; Lee and McCracken 2001). This is surprising given that bats comprise >20% of all mammalian species (Lima and O’Keefe 2013), occurring in colonies that are subject to avian depredation across the globe (Mikula et al. 2016). Owls are the most significant predators of bats in the temperate zones (Speakman 1991), but bat-hunting behavior has been documented in at least 237 species of diurnal birds worldwide (Mikula et al. 2016). Such behavior is often cited as an evolutionary driver of nocturnality in bats (Speakman 1991; Mikula et al. 2016), but empirical evidence is limited, and little is known of the underlying selection pressures (Lima and O’Keefe 2013). In fact, only one previous study has analyzed this behavior from the perspective of group living (Fenton et al. 1994), concluding that the numerical dilution of individual predation risk expected with increasing colony size is partly counteracted by the preference of raptors to hunt at larger colonies, and by variation in the timing and duration of emergence with colony size (Fenton et al. 1994).

**Figure 6:**
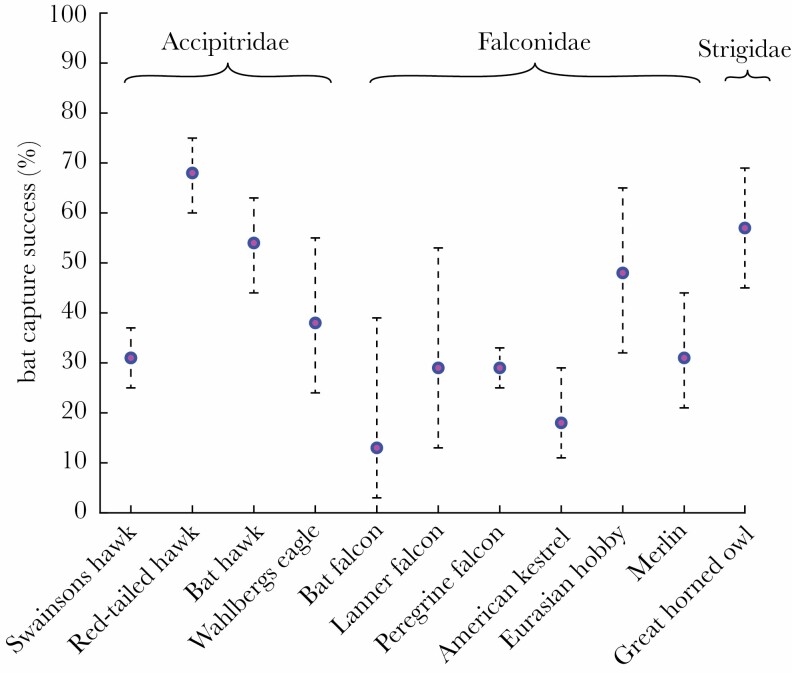
Recorded catch success against bats of 10 species of bird with ≥15 attacks recorded in the literature (Supplementary Table S1), compared with the results of this study for Swainson’s hawks. Error bars display Agresti-Coull 95% CIs.

Swainson’s hawks are opportunistic diurnal hunters that consume a wide variety of mainly terrestrial mammals, reptiles, birds, and insects (Bednarz 1988), but they have been recorded hunting swarming bats in three distinct locations to date (Baker 1962; Harden 1972; Cartron 2010). The range of flexible attack behaviors that we observed in Swainson’s hawks is typical of raptors that hunt bats opportunistically, and the 31% catch success that we observed is comparable to that of other diurnal raptors (Figure 6). Catch success varies according to local conditions, and the much higher 68% catch success reported from red-tailed hawks (*Buteo jamaicensis*) opportunistically hunting another massive maternal colony of Brazilian free-tailed bats may have been skewed by the emergence of juvenile bats (Lee and Kuo 2001). In contrast, our own observations were made prior to the emergence of any juveniles. The very high 54% catch success reported from bat hawks (*Macheiramphus alcinus*) is perhaps more typical, however—this being the only species of bird that is considered morphologically, behaviorally, and ecologically specialized for hunting bats (Jones et al. 2012).

Some of the opportunistic hunting behaviors that we observed in Swainson’s hawks parallel the specialized hunting behaviors of bat hawks, which are reported to intercept bats emerging from their roost in a series of back-and-forth flights (Black et al. 1979), similar to the cross-stream approaches that were prevalent in Swainson’s hawks attacking the column (Figure 3A). Likewise, the Swainson’s hawks that we observed consumed their prey on the wing (see also (Harden 1972; Cartron 2010)), paralleling another well-known behavioral adaptation of bat hawks (Ballance 1981; Auburn 1987; Ansell 1969; Black et al. 1979; Jones et al. 2012). Feeding on the wing is an unusual behavior among raptors, except those like the Eurasian hobby (*Falco subbuteo*) that specialize on smaller aerial prey, but has also been recorded in a peregrine falcon (*Falco peregrinus*) hunting swarming bats (Sprunt 1951). We hypothesize that this behavior is adaptive during mass emergence, when the glut of prey makes it possible to capture multiple bats in a limited time window.

## CONCLUSIONS

Colonial bats run a daily gauntlet against birds as they emerge from their roosts – the localization and repeatability of which makes this an outstanding model system for studying predator-prey interactions in their natural ecological context, and enables us to relate attack outcomes and occurrence to the individual behaviors of predator and prey. This contrasts with previous work on massive three-dimensional prey aggregations, which has focused mainly on the dynamics of the prey’s collective behavior (Carere et al. 2009; Procaccini et al. 2011; Storms et al. 2019); but see (Zoratto et al. 2010), or on the predator’s behavioral response to this (Handegard et al. 2012). Somewhat surprisingly, given the scale and complexity of the visual spectacle, we find no evidence of any confusion effect decreasing hunting efficiency in attacks on the column. Such confusion effects have been demonstrated—though not universally—across a broad range of taxa (Jeschke and Tollrian 2007), including raptors attacking flocking birds (Kenward 1978; Cresswell 1994), for which success rates are usually higher in attacks on lone individuals (Whitfield 2003; Roth et al. 2006; Zoratto et al. 2010). In contrast, the hawks that we studied were no less successful at catching bats in the column. Hence, there is no evidence that the bats benefitted from any vigilance effect either, which is perhaps less surprising given the limited range over which the bats’ echolocation is effective, and given their poor vision compared to many flocking birds in which group vigilance is important (Cresswell 1994; Beauchamp and Ruxton 2008; Beauchamp 2012). Indeed, it is possible that the absence of a confusion effect in this system is coupled to the absence of any vigilance effect, given the lack of clear agitation waves that can result from group evasion and which may also be important in confusing predators (Procaccini et al. 2011).

Even so, lone bats have a significantly higher risk of predation than bats flying within the column. This is because they have a high relative risk of attack—whether as isolated wanderers that do not benefit from dilution through attack abatement (Turner and Pitcher 1986), or as stragglers on the periphery that suffer marginal predation (Duffield and Ioannou 2017)—perhaps coupled with selfish herd effects (Hamilton 1971). Since the hawks did not enjoy a higher success rate against lone bats, their tendency to attack them disproportionately often presumably reflects the fact that predators encounter isolated and marginal individuals first when launching attacks from outside of a group (Duffield and Ioannou 2017), rather than because they specifically target lone individuals to avoid confusion (Quinn and Cresswell 2006). Hence, whereas the fortunes of the prey depend on how successfully they maintain column formation, the fortunes of the predators do not depend on their driving tendency to attack lone prey. Instead, the catch success of the hawks depends on the detailed behavioral tactics that they employ, including stooping dives and rolling grab maneuvers. Given the apparent lack of vigilance by the prey, it seems likely that the success of stooping here has to do with the agility it confers (Mills et al. 2018) and not to do with the element of surprise that may be important against flocking birds (Cresswell 1994; Beauchamp and Ruxton 2008). Since different assemblages of bats and raptors perform similar behaviors at many different locations around the world, it should be possible to test the generality of these conclusions through future comparative study.

## Supplementary Material

araa145_suppl_Supplementary_Movie_S1

araa145_suppl_Supplementary_Information

## Data Availability

Analyses reported in this article can be reproduced using the data provided by Brighton, Caroline (2020). The original video data are archived institutionally, and will be made available by the corresponding author upon reasonable request. Example videos are provided as Supplementary Movie S1.

## References

[CIT0001] Ansell WDH . 1969. A bat hawk (*Macheiramphus alcinus* Anderssoni) at Ngoma, Kafue National Park. Puku. 5:213.

[CIT0002] Auburn J . 1987. RSD and the agility of the bat hawk. Gabar. 2:15–16.

[CIT0003] Axelsen BE , Anker-Nilssen T, Fossum P, Kvamme C, Nottestad L. 2001. Pretty patterns but a simple strategy: predator-prey interactions between juvenile herring and Atlantic puffins observed with multibeam sonar. Can J Zool. 79:1586–1596.

[CIT0004] Baker JK . 1962. The manner and efficiency of raptor depredations on bats. Condor. 64:500–504.

[CIT0005] Ballance TC . 1981. Observations on bat hawk hunting. Honeyguide. 106:29–30.

[CIT0006] Beauchamp G . 2012. Flock size and density influence speed of escape waves in semipalmated sandpipers. Anim Behav. 83:1125–1129.

[CIT0007] Beauchamp G , Ruxton GD. 2008. Disentangling risk dilution and collective detection in the antipredator vigilance of semipalmated sandpipers in flocks. Anim Behav. 75:1837–1842.

[CIT0008] Bednarz JC . 1988. A comparative-study of the breeding ecology of Harris and Swainson hawks in southeastern New Mexico. Condor. 90:311–323.

[CIT0009] Black HL , Howard G, Stjernstedt R. 1979. Observations on the feeding-behavior of the bat hawk (*Macheiramphus alcinus*). Biotropica. 11:18–21.

[CIT0010] Brighton CH , Taylor GK. 2019. Hawks steer attacks using a guidance system tuned for close pursuit of erratically manoeuvring targets. Nat Commun. 10:2462. 31186415 10.1038/s41467-019-10454-z PMC6560099

[CIT0011] Brighton CH , Thomas ALR, Taylor GK. 2017. Terminal attack trajectories of peregrine falcons are described by the proportional navigation guidance law of missiles. Proc Natl Acad Sci USA. 114:13495–13500. 29203660 10.1073/pnas.1714532114 PMC5754800

[CIT0012] Brighton CH , Zusi L, McGowan KA, Kinniry M, Kloepper LN, Taylor GK. 2020. Aerial attack strategies of hawks hunting bats, and the adaptive benefits of swarming. Behav Ecol. doi:10.5061/dryad.k98sf7m51 PMC8177810 34104109

[CIT0013] Cade DE , Carey N, Domenici P, Potvin J, Goldbogen JA. 2020. Predator-informed looming stimulus experiments reveal how large filter feeding whales capture highly maneuverable forage fish. Proc Natl Acad Sci USA. 117:472–478. 31871184 10.1073/pnas.1911099116 PMC6955359

[CIT0014] Canty A , Ripley B. 2019. Boot: Bootstrap R (S-Plus) functions. R package version 1.3–23.

[CIT0015] Carere C , Montanino S, Moreschini F, Zoratto F, Chiarotti F, Santucci D, Alleva E. 2009. Aerial flocking patterns of wintering starlings, *Sturnus vulgaris*, under different predation risk. Anim Behav. 77:101–107.

[CIT0016] Cartron JE . 2010. Raptors of New Mexico. Albuquerque (NM): University of New Mexico Press.

[CIT0017] Cresswell W . 1994. Flocking is an effective anti-predation strategy in redshanks, *Tringa totanus*. Anim Behav. 47:433–442.

[CIT0018] Cresswell W , Quinn JL. 2010. Attack frequency, attack success and choice of prey group size for two predators with contrasting hunting strategies. Anim Behav. 80:643–648.

[CIT0019] Davison AC , Hinkley DV. 1997. Bootstrap methods and their applications. Cambridge: Cambridge University Press.

[CIT0020] Duffield C , Ioannou CC. 2017. Marginal predation: do encounter or confusion effects explain the targeting of prey group edges? Behav Ecol. 28:1283–1292. 29622928 10.1093/beheco/arx090 PMC5873256

[CIT0021] Efron B . 1987. Better bootstrap confidence intervals. J Am Stat Assoc. 82:171–185.

[CIT0022] Fenton MB , Rautenbach IL, Smith SE, Swanepoel CM, Grosell J, Vanjaarsveld J. 1994. Raptors and bats - threats and opportunities. Anim Behav. 48:9–18.

[CIT0023] Foster WA , Treherne JE. 1981. Evidence for the dilution effect in the selfish herd from fish predation on a marine insect. Nature. 293:466–467.

[CIT0024] Gerlotto F , Bertrand S, Bez N, Gutierrez M. 2006. Waves of agitation inside anchovy schools observed with multibeam sonar: a way to transmit information in response to predation. Ices J Mar Sci. 63:1405–1417.

[CIT0025] Gillam EH , Hristov NI, Kunz TH, McCracken GF. 2010. Echolocation behavior of Brazilian free-tailed bats during dense emergence flights. J Mammal. 91:967–975.

[CIT0026] Hamilton WD . 1971. Geometry for the selfish herd. J Theor Biol. 31:295–311. 5104951 10.1016/0022-5193(71)90189-5

[CIT0027] Handegard NO , Boswell KM, Ioannou CC, Leblanc SP, Tjøstheim DB, Couzin ID. 2012. The dynamics of coordinated group hunting and collective information transfer among schooling prey. Curr Biol. 22:1213–1217. 22683262 10.1016/j.cub.2012.04.050

[CIT0028] Harden WD . 1972. Predation by hawks on bats at Vickery Bat Cave. Oklahoma Ornithological Society. 5:4–5.

[CIT0029] Hein AM , Altshuler DL, Cade DE, Liao JC, Martin BT, Taylor GK. 2020. An algorithmic approach to natural behavior. Curr Biol. 30:R663–R675. 32516620 10.1016/j.cub.2020.04.018

[CIT0030] Herreid CF 2nd . 1963. Temperature regulation and metabolism in Mexican freetail bats. Science. 142:1573–1574. 14075688 10.1126/science.142.3599.1573

[CIT0031] Ioannou CC , Guttal V, Couzin ID. 2012. Predatory fish select for coordinated collective motion in virtual prey. Science. 337:1212–1215. 22903520 10.1126/science.1218919

[CIT0032] Ioannou CC , Ramnarine IW, Torney CJ. 2017. High-predation habitats affect the social dynamics of collective exploration in a shoaling fish. Sci Adv. 3:e1602682. 28508069 10.1126/sciadv.1602682 PMC5415332

[CIT0033] Jeschke JM , Tollrian R. 2007. Prey swarming: which predators become confused and why? Anim Behav. 74:387–393.

[CIT0034] Jones LR , Black HL, White CM. 2012. Evidence for convergent evolution in gape morphology of the bat hawk (*Macheiramphus alcinus*) with swifts, swallows, and goatsuckers. Biotropica. 44: 386–393.

[CIT0035] Kenward RE . 1978. Hawks and doves - factors affecting success and selection in goshawk attacks on woodpigeons. J Anim Ecol. 47:449–460.

[CIT0036] Kloepper LN , Linnenschmidt M, Blowers Z, Branstetter B, Ralston J, Simmons JA. 2016. Estimating colony sizes of emerging bats using acoustic recordings. R Soc Open Sci. 3:160022. 27069667 10.1098/rsos.160022 PMC4821278

[CIT0037] Krause J . 1994. Differential fitness returns in relation to spatial position in groups. Biol Rev Camb Philos Soc. 69:187–206. 8054444 10.1111/j.1469-185x.1994.tb01505.x

[CIT0038] Landeau L , Terborgh J. 1986. Oddity and the confusion effect in predation. Anim Behav. 34:1372–1380.

[CIT0039] Lee YF , Kuo YM. 2001. Predation on Mexican free-tailed bats by peregrine falcons and red-tailed hawks. J Raptor Res. 35:115–123.

[CIT0040] Lee YF , McCracken GF. 2001. Timing and variation in the emergence and return of Mexican free-tailed bats, *Tadarida brasiliensis* mexicana. Zool Stud. 40:309–316.

[CIT0041] Lehtonen J , Jaatinen K. 2016. Safety in numbers: the dilution effect and other drivers of group life in the face of danger. Behav Ecol Sociobiol. 70:449–458.

[CIT0042] Lima SL . 1995. Back to the basics of antipredatory vigilance - the group-size effect. Anim Behav. 49:11–20. 10.1006/anbe.1999.1182 10479369

[CIT0043] Lima SL . 2002. Putting predators back into behavioral predator-prey interactions. Trends Ecol Evol. 17:70–75.

[CIT0044] Lima SL , O’Keefe JM. 2013. Do predators influence the behaviour of bats? Biol Rev Camb Philos Soc. 88:626–644. 23347323 10.1111/brv.12021

[CIT0045] Magurran AE , Pitcher TJ. 1987. Provenance, shoal size and the sociobiology of predator-evasion behavior in minnow shoals. Proc R Soc Lond B. 229:439–465.

[CIT0046] Mikula P , Morelli F, Lucan RK, Jones DN, Tryjanowski P. 2016. Bats as prey of diurnal birds: a global perspective. Mamm Rev. 46:160–174.

[CIT0047] Mills R , Hildenbrandt H, Taylor GK, Hemelrijk CK. 2018. Physics-based simulations of aerial attacks by peregrine falcons reveal that stooping at high speed maximizes catch success against agile prey. PLoS Comput Biol. 14:e1006044. 29649207 10.1371/journal.pcbi.1006044 PMC5896925

[CIT0048] Morgan MJ , Godin JGJ. 1985. Antipredator benefits of schooling behavior in a cyprinodontid fish, the banded killifish (*Fundulus diaphanus*). Z Tierpsychol. 70:236–246.

[CIT0049] NOAA solar calculator . 2018. https://www.esrl.noaa.gov/gmd/grad/solcalc/ (accessed 2018).

[CIT0050] Nottestad L , Axelsen BE. 1999. Herring schooling manoeuvres in response to killer whale attacks. Can J Zool. 77:1540–1546.

[CIT0051] Parrish JK . 1989. Reexamining the selfish herd - are central fish safer? Anim Behav. 38:1048–1053.

[CIT0052] Procaccini A , Orlandi A, Cavagna A, Giardina I, Zoratto F, Santucci D, Chiarotti F, Hemelrijk CK, Alleva E, Parisi G et al. 2011. Propagating waves in starling, *Sturnus vulgaris*, flocks under predation. Anim Behav. 82:759–765.

[CIT0053] Quinn JL , Cresswell W. 2006. Testing domains of danger in the selfish herd: sparrowhawks target widely spaced redshanks in flocks. Proc R Soc Lond B. 273:2521–2526. 10.1098/rspb.2006.3612 PMC1634896 16959644

[CIT0054] Rayor LS , Uetz GW. 1990. Trade-offs in foraging success and predation risk with spatial position in colonial spiders. Behav Ecol Sociobiol. 27:77–85.

[CIT0055] R CoreTeam . 2019. R: a language and environment for statistical computing. Vienna (Austria): R Foundation for Statistical Computing.

[CIT0056] Rieucau G , Ferno A, Ioannou CC, Handegard NO. 2015. Towards of a firmer explanation of large shoal formation, maintenance and collective reactions in marine fish. Rev Fish Biol Fisher. 25:21–37.

[CIT0057] Roberts KJ , Yancey FD, Jones C. 1997. Predation by great-horned owls on Brazilian free-tailed bats in north Texas. Texas J. Sci. 49:215–218.

[CIT0058] Rodríguez-Durán A , Lewis AR. 1985. Seasonal predation by merlins on sooty mustached bats in western Puerto Rico. Biotropica. 17:71–74.

[CIT0059] Roth TC , Lima SL, Vetter WE. 2006. Determinants of predation risk in small wintering birds: the hawk’s perspective. Behav Ecol Sociobiol. 60:195–204.

[CIT0060] Scherer R . 2018. PropCIs: various confidence interval methods for proportions. R package version 03-0.

[CIT0061] Similä T . 1997. Sonar observations of killer whales (*Orcinus orca*) feeding on herring schools. Aquat Mamm. 23:119–126.

[CIT0062] Speakman JR . 1991. The impact of predation by birds on bat populations in the British Isles. Mammal Rev. 21:123–142.

[CIT0063] Sprunt A . 1951. Aerial feeding of duck hawk, Falco p. anatum. Auk. 68:372–373.

[CIT0064] Stankowich T . 2003. Marginal predation methodologies and the importance of predator preferences. Anim Behav. 66:589–599.

[CIT0065] Storms RF , Carere C, Zoratto F, Hemelrijk CK. 2019. Complex patterns of collective escape in starling flocks under predation. Behav Ecol Sociobiol. 73:10. 30930523 10.1007/s00265-018-2609-0 PMC6404399

[CIT0066] Turner GF , Pitcher TJ. 1986. Attack abatement - a model for group protection by combined avoidance and dilution. Am Nat. 128:228–240.

[CIT0067] Whitfield DP . 2003. Redshank *Tringa totanus* flocking behaviour, distance from cover and vulnerability to sparrowhawk *Accipiter nisus* predation. J Avian Biol. 34:163–169.

[CIT0068] Wickham H . 2011. The split-apply-combine strategy for data analysis. J Stat Softw. 40:1–29.

[CIT0069] Wrona FJ , Dixon RWJ. 1991. Group-size and predation risk - a field analysis of encounter and dilution effects. Am Nat. 137:186–201.

[CIT0070] Zoratto F , Carere C, Chiarotti F, Santucci D, Alleva E. 2010. Aerial hunting behaviour and predation success by peregrine falcons *Falco peregrinus* on starling flocks *Sturnus vulgaris*. J Avian Biol. 41:427–433.

