# Supplementary material for: Aerial attack strategies of hawks hunting bats, and the adaptive benefits of swarming
Source: Behav Ecol. 2021 Mar 31;32(3):464–76. doi: 10.1093/beheco/araa145 (PMC8177810; doi:10.1093/beheco/araa145)
Supplement: araa145_suppl_Supplementary_Information [file araa145_suppl_supplementary_information.pdf]

Supplementary Information

This file contains:

Figures S1-S2

Tables S1-S3

Supplementary References supporting Table S1

Legend for Movie S1

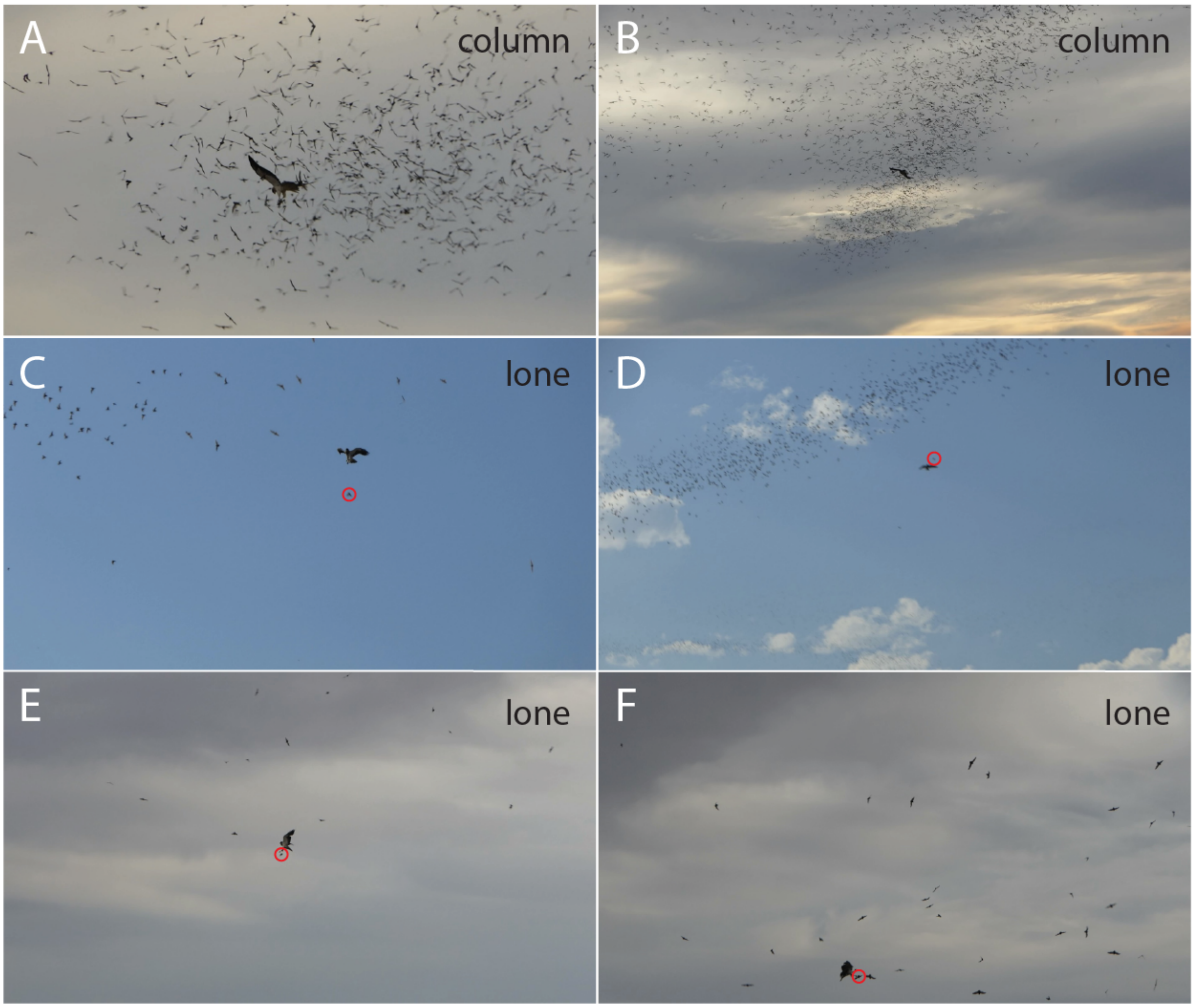

Figure S1. Video frames showing examples of attacks on lone bats and the column. (A,B) Attacks on the column of bats, defined as an attack on one or more bats within a cohesive group of individuals all flying in the same general direction. (C-E) Attacks on a lone bat (circled red), defined as an attack on an individual that appeared to be flying at least 1m from the edge of the column, and typically in a different direction to the swarm. (F) If an attack occurred in a volume containing many bats, but with no coherent flight direction, then this was also categorised as an attack on a lone bat, rather than as an attack on the swarm.

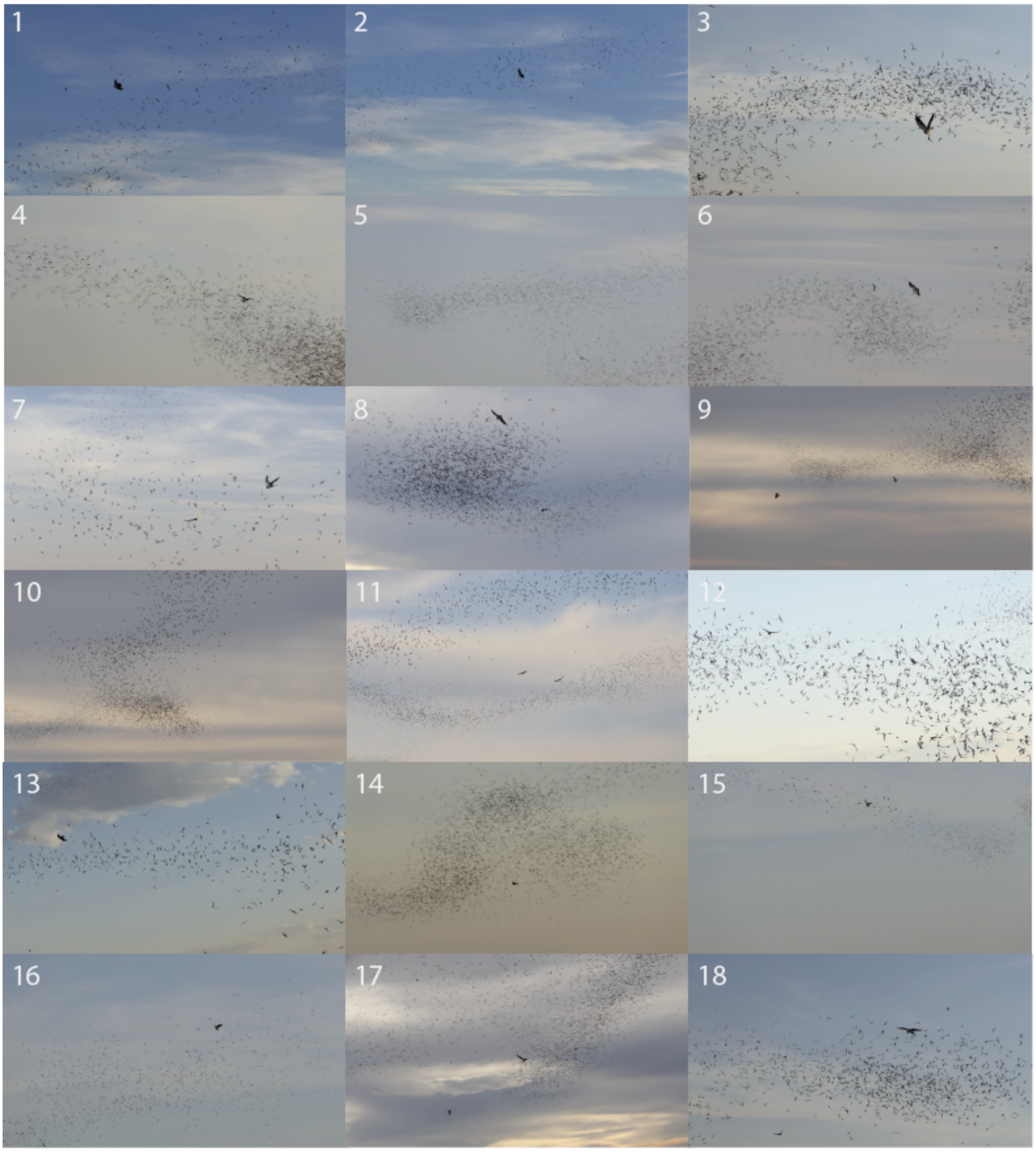

Figure S2 Video frames used to estimate the proportion of bats meeting the criteria for classification as lone bats. These frames were chosen as meeting the following criteria: (i) each frame recorded during a separate attack; (ii) camera zoomed out and in focus; (iii) bats close enough to see their wings; (iv) background composed entirely of sky. See Table S3 for the results of this analysis.

common name	scientific name	stoop or swoop	tail-chase	perch-hunting	hunting lone bat	hunting swarm	dawn	daytime	dusk	cooperative hunting	successes	observations	catch success	lower 95% CI	upper 95% CI	references
**Accipitriformes**																
Bat hawk	*Macheiramphus alcinus*	y	y		y	y			y		59	110	54%	44%	63%	[1-5]
Wahlberg's eagle	*Hieraaetus wahlbergi*	y				y			y		13	34	38%	24%	55%	[6, 7]
Mississippi kite	*Ictinia mississippiensis*	y				y	y		y		-	-				[8]
Double-toothed kite	*Harpagus bidentatus*			y	y	y		y	y		3	4				[9, 10]
Northern harrier	*Circus hudsonius*	y				y					0	-				[11]
Gabar goshawk	*Micronisus gabar*	y		y	y			y	y		1	2				[12, 13]
African goshawk	*Accipiter tachiro*		y	y		y			y		11	14				[6, 14-16]
Cooper's hawk	*Accipiter cooperii*		y		y	y			y		1	1				[10, 17]
Eurasian sparrowhawk	*Accipiter nisus*		y		y	y		y	y		0	10				[18, 19]
Shikra	*Accipiter badius*	y	y	y		y			y		-	-				[20]
Harris's hawk	*Parabuteo unicinctus*			y	y				y		2	-				[21]
Red-tailed hawk	*Buteo jamaicensis*	y		y		y	y		y		96	142	68%	60%	75%	[10, 11, 22-24]
Swainson's hawk	*Buteo swainsoni*	y	y			y			y		7	8				[10, 23]
**Falconiformes**																
Lanner falcon	*Falco biarmicus*	y	y		y	y			y		5	17	29%	13%	53%	[25-27]
Merlin	*Falco columbarius*	y	y	y		y			y		18	58	31%	21%	44%	[28, 29]
Dickinson's kestrel	*Falco dickinsoni*	y		y	y	y			y		10	-				[30, 31]
Red-headed falcon	*Falco chicquera*		y	y		y			y		≥33	-				[32-34]
Peregrine falcon	*Falco peregrinus*	y	y	y	y	y	y	y	y		141	490	29%	25%	33%	[24, 35-38]
Bat falcon	*Falco rufigularis*	y		y	y		y	y	y		2	15	13%	3%	39%	[39, 40]
American kestrel	*Falco sparverius*	y		y	y	y	y	y	y		12	66	18%	11%	29%	[10, 23, 41-43]
Eurasian hobby	*Falco subbuteo*	y	y	y	y	y	y		y		15	31	48%	32%	65%	[6, 44-47]
Australian hobby	*Falco longipennis*	y	y			y			y		3	4				[48]
Sooty falcon	*Falco concolor*		y		y				y		4	0				[49]
Common kestrel	*Falco tinnunculus*	y		y	y	y	y	y	y		137	-				[50-52]
Lesser kestrel	*Falco naumanni*	y	y		y	y	y	y	y		0	≥5				[67]
Australian kestrel	*Falco cenchroides*	y		y	y			y			1	-				[53]
Rock kestrel	*Falco rupicolus*	y				y			y		1	0				[54]
**Strigiformes**																
Barn owl	*Tyto alba*	y		y		y			y		3	4				[11, 42]
Great horned owl	*Bubo virginianus*			y		y			y		36	63	57%	45%	69%	[55-57]
Northern long-eared owl	*Otus asio*			y		y			y		≤5	12				[58]
**Passeriformes**																
Carrion crow	*Corvus corone*			y	y		y				1	2				[59]
Rook	*Corvus frugilegus*		y	y	y				y		0	3				[60]
American crow	*Corvus brachyrhynchos*	y	y		y			y	y	y	2	5				[61, 62]
Large-billed crow	*Corvus macrorhynchos*	y				y	y	y		y	>>1	-				[63]
Black-billed crow	*Pica hudsonia*		y		y			y			1	1				[64]
Great grey shrike	*Lanius excurbitor*			y		y			y		0	≥2				[65]
**Charadriiformes**																
European herring gull	*Larus argentatus*		y		y						1	1				[66]

Table S1. Summary of the results of previous studies recording observations of bat-hunting behaviours in birds. Each of the various categories of hunting behaviours is scored "y" if recorded at least once in that species, and left blank otherwise. A successful attack is defined as an attack in which a bat was caught, regardless of whether it was then eaten. Any study that only reported the number of successful attacks without also stating the total number of attacks observed was excluded from the calculation of catch success. Reported confidence intervals (CIs) are approximate 95% CIs calculated using the Agresti-Coull method after pooling all of the data.

Date	attempted attacks	duration of focal follow (s)	approach type		approach direction			targeting strategy		grab direction			bat captured		wind speed (km h^{-1})	wind direction (degs)
			stooping dive	level flight	down-stream	cross-stream	up-stream	column of bats	lone bat	above	side	below	yes	no		
01/06/2018	12	212	4	8	3	7	2	12	0	4	7	1	2	10	6.6	210
01/06/2018	7	208	0	7	3	NaN	2	6	NaN	NaN	4		1	6	6.6	210
01/06/2018	4	69	NaN	3	1	NaN	NaN	3	1	NaN	NaN	NaN	0	4	6.6	210
01/06/2018	3	75	1	2	2	1	NaN	3	NaN	1	NaN	NaN	0	3	6.6	210
01/06/2018	8	180	1	7	3	5	0	8	0	1	6	1	2	6	6.6	210
02/06/2018	9	552	4	5	NaN	8	NaN	8	1	3	1	NaN	1	8	6.3	230
02/06/2018	8	150	0	8	3	2	NaN	8	0	NaN	5	NaN	1	7	6.3	230
02/06/2018	2	36	0	2	1	NaN	NaN	2	0	NaN	1	NaN	0	2	6.3	230
02/06/2018	2	114	2	0	0	2	0	1	1	1	1	0	1	1	6.3	230
02/06/2018	3	297	1	2	0	3	0	2	NaN	NaN	2	NaN	3	0	6.3	230
04/06/2018	3	175	1	2	1	2	0	3	0	2	1	0	3	0	6.6	230
04/06/2018	7	495	4	3	1	6	0	6	1	1	NaN	6	4	3	6.6	230
04/06/2018	2	97	0	2	0	1	1	1	1	1	1	0	1	1	6.6	230
04/06/2018	5	215	2	2	NaN	3	1	4	NaN	2	1	1	2	3	6.6	230
04/06/2018	4	286	1	1	NaN	1		1	NaN	NaN	NaN	NaN	2	2	6.6	230
04/06/2018	8	28	0	8	0	0	8	8	0	5	3	0	0	8	6.6	230
04/06/2018	3	78	0	3	1	2	0	3	0	0	3	0	1	2	6.6	230
04/06/2018	2	32	0	2	1	1	0	2	0	0	2	0	1	1	6.6	230
04/06/2018	2	164	1	1	0	2	0	2	0	1	1	0	2	0	6.6	230
04/06/2018	5	171	0	5	2	3	0	5	0	2	3	0	1	4	6.6	230
05/06/2018	15	726	2	13	3	11	1	11	4	5	9	1	5	10	8.5	220
05/06/2018	2	28	2	0	0	2	0	2	0	2	0	0	1	1	8.5	220
05/06/2018	2	155	1	1	2	0	0	2	0	1	NaN	NaN	2	0	8.5	220
05/06/2018	1	47	0	1	0	1	0	1	0	0	1	0	1	0	8.5	220
05/06/2018	1	90	0	1	1	0	0	1	0	0	1	0	1	0	8.5	220
05/06/2018	4	259	1	2	NaN	3	NaN	3	NaN	1	2	NaN	3	1	8.5	220
05/06/2018	1	105	0	1	0	0	1	1	0	0	1	0	1	0	8.5	220
05/06/2018	1	205	0	1	0	1	0	1	0	0	1	0	1	0	8.5	220
06/06/2018	1	160	NaN	NaN	NaN	NaN	NaN	NaN	NaN	NaN	NaN	NaN	1	0	33.8	330
06/06/2018	1	75	NaN	NaN	NaN	NaN	NaN	NaN	NaN	NaN	NaN	NaN	1	0	33.8	330
07/06/2018	3	82	0	3	3	0	0	2	1	0	3	0	1	2	9.8	310
07/06/2018	3	120	NaN	2	1	1		3	0		1	1	1	2	9.8	310
07/06/2018	7	20	0	7	3	3	1	7	0	2	5	0	1	6	9.8	310
10/06/2018	7	394	1	6	0	5	2	7	0	0	7	0	4	3	11.4	320
11/06/2018	3	66	1	2	1	2	0	3	0	2	1	0	1	2	15.1	350
11/06/2018	2	115	NaN	1	NaN	1	NaN	1	NaN	NaN	1	NaN	2	0	15.1	350
11/06/2018	3	49	0	3	0	3	0	3	0	0	2	1	0	3	15.1	350
12/06/2018	3	85	1	2	0	2	1	3	0	1	2	0	1	2	13.2	210
18/06/2018	3	36	0	3	0	3	0	3	0	1	2	0	2	1	14.6	150
18/06/2018	5	197	0	5	0	5	0	5	0	3	2	0	1	4	14.6	150
18/06/2018	2	40	0	2	1	1	0	2	0	1	1	0	0	2	14.6	150
18/06/2018	4	235	1	3	1	2	1	4	0	2	2	0	2	2	14.6	150
19/06/2018	3	180	1	2	0	3	0	3	0	1	1	1	1	2	14.6	140
19/06/2018	1	30	0	1	0	1	0	0	1	0	1	0	0	1	14.6	140
19/06/2018	1	107	0	1	0	1	0	1	0	0	1	0	0	1	14.6	140
19/06/2018	9	439	1	8	5	4	0	4	4	5	3	NaN	1	8	14.6	140
21/06/2018	2	201	1	1	0	2	0	2	0	1	1	0	2	0	3.9	120
21/06/2018	5	231	0	5	1	4	0	5	0	3	2	0	0	5	3.9	120
22/06/2018	1	30	0	1	0	1	0	1	0	0	1	0	0	1	14.3	220
22/06/2018	4	108	0	4	0	4	0	1	3	2	2	0	1	3	14.3	220
22/06/2018	6	75	0	6	1	5	0	6	0	2	3	1	0	6	14.3	220
22/06/2018	3	60	0	3	NaN	1	NaN	1	2	1	2	0	0	3	14.3	220
23/06/2018	1	119	1	0	0	1	0	1	0	1	0	0	1	0	16.3	140
23/06/2018	9	122	7	2	2	7	0	7	2	5	1	NaN	1	8	16.3	140
23/06/2018	2	240	1	1	1	1	0	2	0	1	1	0	1	1	16.3	140
23/06/2018	1	250	0	1	0	1	0	1	0	1	0	0	1	0	16.3	140
23/06/2018	3	120	1	2	0	3	0	3	0	1	2	0	0	3	16.3	140
23/06/2018	1	480	0	1	0	1	0	1	0	1	0	0	1	0	16.3	140
23/06/2018	7	108	3	4	1	4	2	6	1	6	1	0	1	6	16.3	140
23/06/2018	1	145	1	0	0	1	0	1	0	0	1	0	1	0	16.3	140
23/06/2018	1	123	0	1	1	0	0	1	0	0	1	0	1	0	16.3	140
24/06/2018	1	20	0	1	1	0	0	1	0	0	1	0	0	1	11.4	220
24/06/2018	2	60	0	2	0	2	0	2	0	1	0	1	0	2	18.7	320
24/06/2018	2	57	0	2	2	0	0	2	0	0	2	0	0	2	18.7	320
**total**	**239**	**10228**	**49**	**181**	**53**	**142**	**23**	**205**	**23**	**77**	**113**	**15**	**74**	**165**		
**% of total**			**21.3**	**78.7**	**24.3**	**65.1**	**10.6**	**89.9**	**10.1**	**37.6**	**55.1**	**7.3**	**31.0**	**69.0**		
**95% CI (%)**			**16.2<br>27.4**	**72.6<br>83.8**	**19.1<br>30.4**	**58.6<br>71.1**	**7.1<br>15.3**	**85.3<br>93.2**	**6.8<br>14.7**	**31.2<br>44.4**	**48.3<br>61.8**	**4.5<br>11.7**	**25.4<br>37.1**	**62.9<br>74.6**		

Table S2. Classification of hawk attack flights against bats, from observational data. Orange highlights all observations made using the binocular follows, and grey shows observations made using video cameras. A 'NaN' denotes missing data. NB 'hawk no.' only highlights sequential hawks observed on a given night, but we could not identify these same hawks from day-to-day.

frame	binarization threshold	total bat count	lone bat count	proportion of lone bats
1	93	1137	7	0.62%
2	110	868	3	0.35%
3	133	1270	3	0.24%
4	145	1558	2	0.13%
5	140	2682	4	0.15%
6	140	1874	3	0.16%
7	154	702	2	0.28%
8	131	2568	3	0.12%
9	115	2168	2	0.09%
10	125	2687	5	0.19%
11	160	3576	4	0.11%
12	213	1417	3	0.21%
13	103	1234	7	0.57%
14	132	3778	2	0.05%
15	139	777	3	0.39%
16	131	924	4	0.43%
17	111	3404	4	0.12%
18	119	1312	6	0.46%
**total**		**33936**	**67**	**0.20%**

Table S3. Proportion of bats meeting the criteria for classification as lone bats in the 18 video frames in Fig. S2. Bats meeting the criteria for classification as lone bats were counted individually in each frame, and compared to the total number of bats estimated using the object count function in Adobe Photoshop CC2019, after binarizing each image using a binarization threshold just sufficient to make the background sky entirely white. Because individuals with overlapping silhouettes are counted as a single object, this method results in a highly conservative estimate of the proportion of individuals meeting the criteria for classification as lone bats in each frame.

Supplementary References

1. Ansell, W.D.H. A bat hawk (*Macheiramphus alcinus anderssoni*) at Ngoma, Kafue National Park. *The Puku* 1969. **5**: 213-214.
2. Black, H.L., Howard, G., and Stjernstedt, R. Observations on the Feeding-Behavior of the Bat Hawk (*Macheiramphus alcinus*). *Biotropica*, 1979. **11**(1): 18-21.
3. Auburn, J. RSD and the agility of the Bat Hawk. *Gabar*, 1987. **2**: 15-16.
4. Ballance, T.C. Observations on Bat Hawk hunting. *Honeyguide*, 1981. **106**: 29-30.
5. Eccles, D.H., Jensen, R.A.C., and Jensen, M.K. Foraging behaviour of the bat hawk. *Ostrich*, 1969. **40**: 26-27.
6. Fenton, M.B., *et al.* Raptors and bats: threats and opportunities. *Animal Behaviour*, 1994. **48**(1): 9-18.
7. Stephens, W.B. and Blackwood, V. Wahlberg's Eagle catching bats in Malawi. *Ostrich*, 1993. **54**: 25.
8. Taylor, J. Noteworthy Predation on the Guano Bat. *Journal of Mammalogy*, 1964. **45**(2): 300-301.
9. Boinski, S., and Timm, R.M. Predation by squirrel monkeys and double-toothed kites on tent-making bats. *American Journal of Primatology*, 1985. **9**: 121-127.
10. Baker, J.K. The manner and efficiency of raptor depredations on bats. *The Condor*, 1962. **64**(6): p500-504.
11. Looney, M.W. Predation on bats by hawks and owls. *Bulletin of the Oklahoma Ornithological Society*, 1972. **5**(1): 1-5.
12. McGrew, W.C., Gabar goshawk drowns its prey. *Ostrich*, 1980: **51**(1): 53.
13. Mikula, P.H. and Hromada, M., An observation of successful bat predation by Gabar Goshawk *Micronisus gabar* at Ndoto Mountains, Kenya. *Scopus* 2015. **35**(1): 51-52.
14. Cyrus, D. African Goshawk hunting bats at dusk and at midday. *Bokmakierie*, 1983. **35**: 23-24.
15. van Jaarsveld, J. African Goshawks and European Hobbies bat-hawking. *Gabar*, 1988. **3**: 29-31.
16. Kemp, A.C., Rautenbach, I. L. Bat hawks or bat-eating hawks? *Gabar*, 1987. **2**: 4-6.
17. Leopold, A.S. Cooper's Hawk Observed Catching a Bat. *The Wilson Bulletin*, 1944. **56**(2): 116.
18. Borowski, S. Sparrow hawk hunting on the bats. *Notatki Ornitologiczne*, 1968. **9**: 40-41. (in Polish)
19. Roworth, P.C. and Wright, E. Sparrowhawk attacking noctule bats. *British Birds*, 1989. **82**: 564-565.
20. Pettet, A. Shikra *Accipiter badius* taking bats. *Bulletin of the Nigerian Ornithological Society*, 1976. **12**(41): 42.
21. Ortega-Álvarez, R. and Calderón-Parra, R. Hunting the unexpected: Harris's Hawks (*Parabuteo unicinctus*) preying on bats in a Neotropical megacity. *Revista Brasileira De Ornitologia*, 2014. **22**(3): 297-299.
22. Macy, R.M. and Macy, R.W. Hawks as enemies of bats. *Journal of Mammalogy*, 1939. **20**(2): 252.
23. Harden, W.D. Predation by hawks on bats at Vickery Bat Cave. *Bulletin of the Oklahoma Ornithological Society*, 1972. **5**(1): 4-5.
24. Lee, Y.F. and Kuo Y.M. Predation on Mexican free-tailed bats by Peregrine Falcons and Red-tailed Hawks. *Journal of Raptor Research*, 2001. **35**(2): 115-123.
25. Yosef, R. Foraging habits, hunting and breeding success of Lanner Falcons (*Falco biarmicus*) in Israel. *Journal of Raptor Research*, 1991. **25**(3): 77-81.
26. Thomsett, S. Bat hunting by Lanner Falcons in Kenya. *Gabar*, 1987. **2**: 7-8.
27. Laycock, P. Avian predation on cave-dwelling insectivorous bats. *Bokmakierie*, 1982. **34**(1): 17-18.
28. Martinez, S.G. and Lee T.E. Predation on Mexican Free-Tailed Bats (*Tadarida brasiliensis*) by Merlin (*Falco columbarius*). *Southwestern Naturalist*, 2013. **58**(4): 508-512.
29. Rodríguez-Durán, A. and Lewis, A.R. Seasonal predation by Merlins on Sooty Mustached Bats in Western Puerto Rico. *Biotropica*, 1985. **17**(1): 71-74.
30. Hanmer, D.B. and Blackwood, J.G.V. A bat-eating kestrel. *Ostrich*, 1982. **53**: 188-189.
31. Bennet, G. Editor's comment on: Rock Kestrel takes bat (Meinesz, A. and Meinesz, S.). *Bokmakierie*, 1982. **34**(2): 41.
32. Foysal, M. Observations of Red-headed Falcon *Falco chicquera* (Aves: Falconiformes: Falconidae) nest at Keraniganj, Dhaka, Bangladesh, with a focus on post-fledging behavior. *Journal of Threatened Taxa*, 2015. **7**(5): 7138-7145.
33. Fry, C.H. Red-necked Falcon *Falco chicquera* hunting bats. *Bulletin of the Nigerian Ornithological Society*, 1964. **1**: 19.
34. Hanmer, D.B. Red-necked falcons hunting bats. *Bokmakierie*, 1982. **35**(1): 24.
35. Pereira, G.A., *et al.* Occurrence and feeding habits of Peregrine Falcon *Falco peregrinus* in the State of Pernambuco, Brazil. *Revista Brasileira de Ornitologia*, 2006. **14**(4): 435-439. (in Portuguese).
36. Sick, H. Peregrine Falcon hunting bats while wintering in Brazil. *Auk*, 1961. **78**(4): 646-648.
37. Stager, K.E. A group of bat-eating duck hawks. *Condor*, 1941. **43**: 137-139.

38. Byre, V.J., A group of young Peregrine falcons prey on migrating bats. *Wilson Bulletin*, 1990. **102**: 728-730.
39. Robinson, S.K., Habitat selection and foraging ecology of raptors in Amazonian Peru. *Biotropica*, 1994. **26**(4): 443-458.
40. Tostain, O. Adaptation du mode de chasse chez le faucon des chauves-souris (*Falco rufigularis*) en Guyane. *Alauda*, 1986. **54**: 66-67.(in French)
41. Lenoble, A., *et al*. Predation of Lesser Naked-Backed Bats (*Pteronotus Davyi*) by a pair of American Kestrels (*Falco sparverius*) on the island of Marie-Galante, French West Indies. *Journal of Raptor Research*, 2014. **48**(1): 78-81.
42. Twente, J.W. Predation on bats by hawks and owls. *Wilson Bulletin*, 1954. **66**: 135-136.
43. Moura de Souza Aguiar, L., Motta, A., and Esberárd, C., *Falco sparverius* (Aves: Falconiformes) preying upon *Nyctinomops laticaudatus* (Chiroptera: Molossidae). *Zoologia (Curitiba)*, 2012. **29**(2): 180-182.
44. Stanton, D.J, Predation of Dawn-Swarming Bats by Eurasian Hobby (*Falco subbuteo*). *Journal of Raptor Research*, 2016. **50**(3): 317-319.
45. Dronneau, C. and Wassmer, B. Behaviour of juvenile Hobby *Falco subbuteo* after fledging. *Alauda*, 2005. **73**: 33-52. (in French)
46. Dronneau, C. and Wassmer, B. Feeding ecology and hunting behaviour of European Hobby *Falco subbuteo* in eastern France (Alsace). Alauda, 2008. **76**(2): 113-134. (in French)
47. Paci, A.M. Un altro caso di predazione su Chirotteri da parte del Lodolaio (*Falco subbuteo*). Uccelli d'Italia, 1997. **22**(1): 64. (in Italian)
48. Czechura, G.V. Predation on a small colony of bats by an Australian hobby, *Falco longipennis*. *The Victorian Naturalist*, 1983. **98**: 200-203.
49. Sinclair, I., Young, D. and Mowat, C. Sooty falcons feeding on bats. *Albatross – Newsletter of the Natal Bird Club*. 1982. **267**.
50. Tout, P. Kestrel regularly catching bats. *British Birds*, 1986. **79**: 431-432.
51. Negro, J.J., Íbañez, C., Pérez Jordá, J.L., and De La Riva, M.J. Winter Predation by Common Kestrel *Falco tinnunculus* on Pipistrelle Bats *Pipistrellus pipistrellus* in Southern Spain. *Bird Study*, 1992. **39**: 195-199.
52. Mikula, P., Hromada, M., and Tryjanowski, P. Bats and Swifts as food of the European Kestrel (*Falco tinnunculus*) in a small town in Slovakia. *Ornis Fennica*, 2013. **90**(3): 178-185.
53. Lewis, M.J. Australian Kestrels *Falco cenchroides* Feeding on Bats. *Australian Bird Watcher*, 1987. **12**(4): 126-127.
54. Meinesz, A. and Meinesz, S. Rock Kestrel takes a bat. *Bokmakierie*, 1982. **34**(2): 40-41.
55. Constantine, D.G. Great bat colonies attract predators. *Bulletin of the National Speleological Society*, 1948. **10**: 100.
56. Caire, W. and Ports, M. An adaptive method of predation by *Bubo virginianus* (Strigiformes: Strigidae) on Mexican free-tailed bats (Chiroptera: Mollosidae). *The Southwestern Naturalist*, 1981. **26**(1): 69-70.
57. Roberts, K.J, .Yancey, F.D., and Jones, C., Predation by great-horned owls on Brazilian free-tailed bats in North Texas. *The Texas Journal of Science*, 1997. **49**(3): 215-218.
58. Barclay, R.M.R., Thomson, C.E., and Phelan, F. J. S. Screech owl *Otus asio* attempting to capture little brown bats *Myotis lucifugus* at a colony. *Canadian Field-Naturalist*, 1982. **96**: 205-206.
59. Arnold, M.A. Bat as the prey of a Carrion Crow. *British Birds*, 1955. **48**: 91.
60. Radford, A.P. Rooks chasing small bats. *British Birds*, 1984. **77**(3): 119-120.
61. Hernández, D.L., Mell, J.J., and Eaton, M.D., Aerial predation of a bat by an American Crow. *Wilson Journal of Ornithology*, 2007. **119**(4): 763-764.
62. Lefevre, K.L., Predation of a Bat by American Crows, *Corvus brachyrhynchos*. Canadian Field-Naturalist, 2005. **119**: 443-444.
63. Tanalgo, K.C., Waldien, D.L., Monfort, N., and Hughes, A.C., Attacked from above and below, new observations of cooperative and solitary predators on roosting cave bats. *bioRxiv*, 2019. 550582, https://doi.org/10.1101/550582
64. Hochachka, W.M. and Scharf, C. S., Black-billed Magpie, *Pica pica*, predation on bats. *Canadian Field-Naturalist*, 1986. **100**(1): 121-122.
65. Gorman, G., Great Grey Shrike (*Lanius excubitor*) hunting Noctule Bats. *Aquila*, 1998. **153**: 103-104.
66. Cleeves, T.R., Herring gull catching and eating bat. *British Birds*, 1969. **62**: 333.
67. Paterson, A.M. Lesser Kestrel hunting bats. *British Birds*, 1991. **84**(4): 151.

Movie S1. Example video sequences illustrating the behavioural classifications used to describe attacks by Swainson's hawks on Brazilian free-tailed bats. See Box 1 for definitions of the various categories of behaviour. This video is compiled from clips recorded in a mixture of HD (1920×1080 pixels; 50 fps) and 4K UHD (3840×2160 pixels; 25 fps), uniformly downsampled to 1920×1080 pixels at 25 fps using H.264 compression.
